# The evolution of relapse of adult T cell acute lymphoblastic leukemia

**DOI:** 10.1186/s13059-020-02192-z

**Published:** 2020-11-23

**Authors:** Inés Sentís, Santiago Gonzalez, Eulalia Genescà, Violeta García-Hernández, Ferran Muiños, Celia Gonzalez, Erika López-Arribillaga, Jessica Gonzalez, Lierni Fernandez-Ibarrondo, Loris Mularoni, Lluís Espinosa, Beatriz Bellosillo, Josep-Maria Ribera, Anna Bigas, Abel Gonzalez-Perez, Nuria Lopez-Bigas

**Affiliations:** 1 Institute for Research in Biomedicine (IRB Barcelona), The Barcelona Institute of Science and Technology, Barcelona, Spain; 2 Barcelona Institute of Science and Technology (BIST), Baldiri i Reixac 10, 08028 Barcelona, Spain; 3 Hematology Departments, ICO-Hospital Germans Trias i Pujol, Josep Carreras Research Institute, Universitat Autònoma de Barcelona, Badalona, Spain; 4 Program in Cancer Research, Institut Hospital del Mar d’Investigacions Mèdiques, CIBERONC, Barcelona, Spain; 5 Pathology Department, CIBERONC, Hospital del Mar, IMIM, Barcelona, Spain; 6 CMR[B] Center of Regenerative Medicine, Barcelona, Spain; 7 Research Program on Biomedical Informatics, Universitat Pompeu Fabra, Barcelona, Spain; 8 Institució Catalana de Recerca i Estudis Avançats (ICREA), Barcelona, Spain

**Keywords:** T-ALL, Adult acute lymphoblastic leukemia, T-ALL evolution under therapy, Evolution of leukemia relapse, ALL relapse

## Abstract

**Background:**

Adult T cell acute lymphoblastic leukemia (T-ALL) is a rare disease that affects less than 10 individuals in one million. It has been less studied than its cognate pediatric malignancy, which is more prevalent. A higher percentage of the adult patients relapse, compared to children. It is thus essential to study the mechanisms of relapse of adult T-ALL cases.

**Results:**

We profile whole-genome somatic mutations of 19 primary T-ALLs from adult patients and the corresponding relapse malignancies and analyze their evolution upon treatment in comparison with 238 pediatric and young adult ALL cases. We compare the mutational processes and driver mutations active in primary and relapse adult T-ALLs with those of pediatric patients. A precise estimation of clock-like mutations in leukemic cells shows that the emergence of the relapse clone occurs several months before the diagnosis of the primary T-ALL. Specifically, through the doubling time of the leukemic population, we find that in at least 14 out of the 19 patients, the population of relapse leukemia present at the moment of diagnosis comprises more than one but fewer than 10^8^ blasts. Using simulations, we show that in all patients the relapse appears to be driven by genetic mutations.

**Conclusions:**

The early appearance of a population of leukemic cells with genetic mechanisms of resistance across adult T-ALL cases constitutes a challenge for treatment. Improving early detection of the malignancy is thus key to prevent its relapse.

**Supplementary information:**

**Supplementary information** accompanies this paper at 10.1186/s13059-020-02192-z.

## Background

Acute lymphoblastic leukemia (ALL) affects 3 children in 100,000 in the UK [1]. In the past 5 decades, intense research on this disease has succeeded in reducing the mortality of ALL-affected children by 82% [2]. Recently, with the development of cancer genomics, researchers have unraveled the most frequent somatic genetic alterations underlying its development [3–14], and molecular subtypes, as well as their clinical relevance [15–22]. Genetic alterations that elicit some relapse events have also been uncovered, and the potential role of therapy in the development of such relapse cases has been explored [23–31].

ALL is less prevalent in adults (0.7 patients in 100,000 people [1]). Not only are there differences in incidence among age groups, but also relapses after treatment appear more frequently in adults (40–75% vs 15–20% among pediatric patients) [31]. Very few studies have been dedicated to understanding the genomic roots of the emergence of adult ALL, and in particular, of T cell ALL (T-ALL) [32–36]. There is a larger gap in the study of the evolution of this malignancy under therapy and its relapse after treatment. Therefore, important questions regarding the genomic evolution of adult T-ALL remain unanswered. It is not entirely clear, for example, whether the same mutational processes are involved in the onset of pediatric and adult T-ALL cases, and if the chemotherapeutic drugs employed in the treatment leave a mutational footprint in relapse cells, as it has been shown for pediatric cases [37]. Furthermore, while some genetic mechanisms of resistance to treatment have been identified in pediatric ALL [26, 27], it is not known whether these also contribute to resistance of the adult malignancy.

To explore the evolution of adult T-ALL under treatment and address these specific questions, we profiled the whole-genome somatic mutations of 19 T-ALLs from adult patients who relapsed after treatment (in-house cohort; Additional file 1: Table S1). Samples were taken at the time of diagnosis (primary) and at recurrence of the malignancy after treatment (relapse). We then analyzed the genomic evolution of these adult T-ALL cases in comparison with 238 pediatric and young adult ALL cases (71 with primary and relapse samples) available in the public domain (Table 1). Known or potential resistance mutations appear in 6 patients of the cohort. Nevertheless, our results show that in the 19 cases the relapse is driven by genetic mutations, and that resistant cells appear in the population of blasts several months before the diagnosis of the primary.

**Table 1:** Summary of ALL cohorts analyzed

ALL subtype cohort name	Subtype cohort information	Source^^^	Sequencing	Type	Num. patients
DUX4-ERG	Rearrangement and overexpression of DUX4 and transcriptional deregulation or deletion of the transcription factor gene ERG	St. Jude	WGS	B-ALL	30
Infant MLL-R	Infant patients with a fusion of the N-terminus of the MLL gene with the C-terminus of a partner gene	St. Jude	WGS	B-ALL	21
Ph positive	Patients with the “Philadelphia” chromosome present a translocation of chromosomes 9 and 22. This translocation creates the BCR-ABL fusion	St. Jude	WGS	B-ALL	11
Ph-like	Cell gene expression profile of the lymphoblasts of Ph-like ALL is similar to that of Ph positive ALL; however, they do not present BCR-ABL1 rearrangement	St. Jude	WGS	B-ALL	18
Hyperdiploid	Hyperdiploid patients are characterized by multiple chromosomal gains	St. Jude	WGS	B-ALL	40
Hypodiploid	Hypodiploid patients are characterized by chromosomal losses	St. Jude	WGS	B-ALL	22
iAMP21	Patients with intrachromosomal amplification of chromosome 21	St. Jude	WGS	B-ALL	12
T-ALL Zhang	Patients with T cell ALL from Zhang et al. [5] *Nat Gen*	St. Jude	WGS	T-ALL	13
T-ALL Oshima	Patients with T cell ALL from Oshima et al. [30] *PNAS*	Columbia University	WXS	T-ALL	31*
B-ALL Oshima	Patients with B cell ALL from Oshima et al. [30] *PNAS* (B cell lineage subtype unspecified)	Columbia University	WXS	B-ALL	24*
T-ALL Li^#^	Patients with T cell ALL from Li et al. [37] *Blood*	St. Jude	WGS	T-ALL	16*
T-ALL in-house	In-house cohort	In-house	WGS	T-ALL	19*

*WGS* whole-genome sequencing, *WXS* whole-exome sequencing

*Cohorts with primary and relapsed paired samples

^#^Mutations called by the authors of the original analysis; in all other cohorts, a uniform mutation calling pipeline was applied

^^^Source: St. Jude cohorts were defined according to their ALL subtype in different publications (see the “Methods” section) except for the T-ALL pediatric cohort from Li et al. [37]

## Results

### The genomics of primary adult T-ALL

**Fig. 1:**
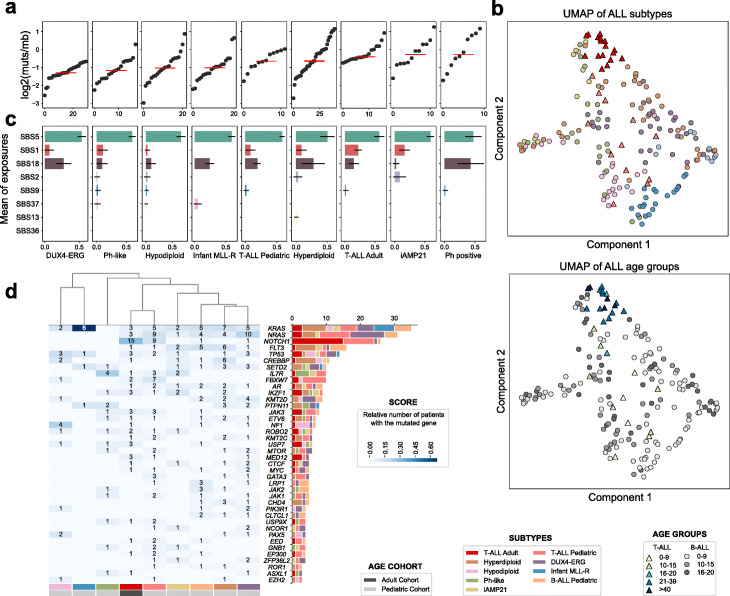
Comparison of primary adult and pediatric ALL cases. **a** Clonal mutation burden (per megabase) of primary T-ALLs of nine cohorts. Red lines represent the median mutation burden of tumors in each cohorts. Tumors are represented as dots, sorted along the *x*-axis according to their mutation burden. **b** Mutational profiles of primary ALLs in the nine cohorts in a uniform manifold approximation and projection (UMAP) dimensionality reduction graph (see the “Methods” section). The UMAP was run on a matrix of the counts of all possible tri-nucleotide changes (96) across ALL patients of all cohorts. Each dot represents a patient, colored according to the cohort (top panel) or their age (bottom panel). **c** Mutational processes active across primary ALL cohorts, represented by their mean (and standard deviation) contribution of the mutation burden of each cohort. SBS1, SBS5, SBS2, SBS9, SBS37, SBS13, SBS36, respectively, single nucleotide substitutions signatures 1,5,2,9,37,13,36. **d** Rate of mutations of selected frequently mutated cancer genes across primary T-ALL cohorts. Cohorts are clustered according to the similarity in their profile of cancer genes mutation frequency (see the “Methods” section). The total number of patients in each cohort with mutations of each cancer gene is represented by bars at the right side of the graph. Only genes with mutations in at least two patients are included in the plot (for the full list see Additional file 2: Fig. S2 and Additional file 1: Tables S3 and S4)

Previous studies on the genomic basis of pediatric ALL have identified somatic mutations across cohorts of patients suffering from this disease [5–8, 10, 12, 13, 28–30, 38–41]. Therefore, we first aimed to compare the landscape of somatic alterations observed across primary adult T-ALL with that across eight other cohorts of T- and B-ALL patients of varying age, ranging from infancy to young adulthood, which we analyzed with a unified mutation calling approach (Table 1; Additional file 1: Tables S1 and S2). Among cancer types, ALL presents a relatively low somatic mutation burden [42, 43]. Nevertheless, the burden of somatic point mutations of adult ALL cases tends to be higher than that of cases of most of the subtypes of the pediatric malignancy, as has been previously observed [44] (Fig. 1a).

Mutations in human somatic cells are contributed to by different molecular mechanisms involving the interaction of endogenous (for instance, spontaneous cytosine deamination or oxidative damage) and external DNA damaging agents (such as UV-light, tobacco carcinogens, or chemotherapies) with the DNA repair machinery [42, 45–47]. The study of these mutational processes in tumors reveals the lifetime exposures of patients to potential carcinogenic agents and consequently contributes to shedding light on the etiology of malignancies. Thus, we first asked whether the somatic mutations observed across nine cohorts of pediatric and adult ALL (Table 1) are contributed by similar or different mutational processes. No clear differences are observed between the mutational profiles of B-ALL and T-ALL (Fig. 1b, top). However, the mutational profiles of pediatric and adult malignancies exhibit discernible, albeit slight differences (Fig. 1b, bottom). The same mutational processes appear to be active across pediatric and adult T-ALL and in pediatric B-ALL (Fig. 1c; Additional file 2: Fig. S1). In particular, mutational signature 5 (SBS5), which in blood has been demonstrated to behave in a clock-like manner [48], and has been associated with the process of hematopoietic cell divisions [49, 50], appears as one of the main contributors of mutations in the evolution of both pediatric and adult ALL.

We next asked whether the driver alterations observed across primary adult T-ALL in the in-house cohort are different from those observed across pediatric B/T-ALL (see the “Methods” section; Fig. 1d; Additional file 2: Fig. S2; Additional file 1: Tables S3 and S4). Mutations in some known ALL driver genes, such as NOTCH1 and FBXW7 (the E3-ligase charged with its recognition for ubiquitination [51]), are overrepresented among both pediatric and adult T-ALL in comparison with B-ALLs (*χ*^2^ *p* = 1.05 × 10^−16^ and *χ*^2^ *p* = 8.37 × 10^−9^, respectively). Similar overrepresentation of mutations in T-ALLs was found in JAK3 (*χ*^2^ *p* = 0.004). In contrast, RAS activating mutations do not appear to be differently represented in both ALL types (*χ*^2^ *p* = 0.05 and *χ*^2^ *p* = 0.634 for KRAS and NRAS).

### Genomic alterations driving primary and relapse adult T-ALL

**Fig. 2:**
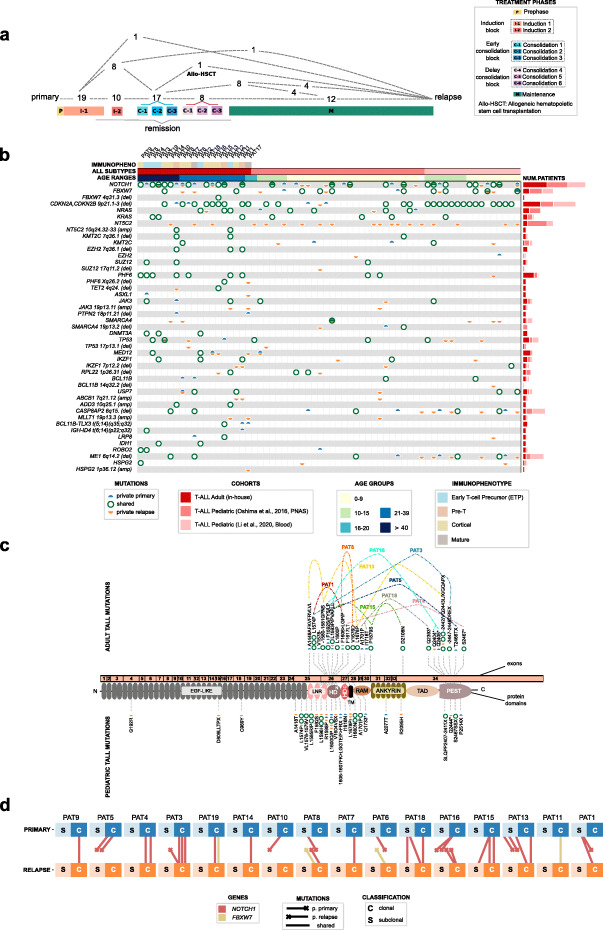
Comparison of different age groups in T cell acute lymphoblastic leukemia. **a** Schematic representation of the clinical course of all patients in the in-house T-ALL cohort. Colored boxes (following the legend) at the bottom depict common stages in this clinical course. The broken lines represent specific trajectories followed by groups of patients, with the numbers in each trajectory. **b** Summary of driver mutations (single nucleotide variants, indels, copy number variants and translocations) identified in the primary and/or relapse T-ALLs of adult and pediatric patients. The original cohorts and age bins of the patients included in the table are indicated above it. The sample where the mutation is identified (primary, relapse, or both) is indicated by color semicircles and circles at each cell of the table. The total number of patients affected by mutations of each gene is indicated by the bars at the right-side of the graph. The table contains the genes that are altered in at least two patients of the adult cohort (for full table see Additional file 2: Fig. S4 and Additional file 1: Table S5). **c** Protein affecting mutations identified in NOTCH1 gene within adult (above graph) and pediatric (below graph) T-ALLs. Multiple mutations in different samples of a patient in the in-house are represented as dashed colored lines that connect the mutated positions. (No line connects mutations in patients of pediatric cohorts, even if multiple mutations affecting NOTC1 in a patient were observed.) **d** Clonality change in multi-mutated NOTCH1 pathway genes. Blue and orange squares depict, respectively, primary and relapse T-ALL samples of each patient. Lines connecting them represent shared (connecting lines) or private (lines ending in a cross) NOTCH1 or FBXW7 mutations. In 5 out of 19 patients, only one mutation in this pathway is identified, while in the other 11, multiple mutations are detected. We did not detect any mutation affecting this pathway in only 3 of the 19 patients

With the goal to study the evolution of adult T-ALL, the 19 patients in the in-house cohort were selected specifically because they relapsed several months after treatment (Fig. 2a; Additional file 2: Fig. S3; Additional file 1: Table S1). Seventeen of them received the same treatment protocol (ALL-HR-11 [NCT01540812]), while the remaining two were administered very similar protocols (LAL-07OLD and ALL-HR-2003 [NCT00853008]). To uncover the genomic similarities and differences between adult and pediatric T-ALL cases at relapse, we next compared the in-house cohort with 31 relapsed cases from the T-ALL Oshima and T-ALL SJ cohorts (Table 1; Additional file 1: Tables S3 and S4). A list of potential driver events across the 19 patients in the cohort is presented in Additional file 1: Tables S5 and S6.

Many NOTCH1 and FBXW7 mutations observed in the primary leukemias were also present in the relapse samples (Fig. 2b; Additional file 2: Fig. S4). Intriguingly, mutations affecting USP7, a known deubiquitinase of NOTCH1, were detected in 3 adult and 3 pediatric patients, raising the possibility of yet another form of alteration of the NOTCH pathway in leukemogenesis [52–54]. Overall, NOTCH1-affecting mutations in adults are distributed along the protein-coding sequence in a very similar manner as those observed in pediatric patients (Fig. 2c). Nine patients in the cohort present multiple mutations of NOTCH1 that affect different protein domains (mostly HD and PEST), in agreement with previous reports [55]. Interestingly, in 6 patients, different NOTCH1/FBXW7 mutations were detected in the primary and relapse samples (Fig. 2d). These constitute examples of convergent evolution of mutations affecting the NOTCH1 pathway, also observed in eight pediatric patients in the cohorts analyzed. This suggests that NOTCH1 mutations tend to appear late [56] and recurrently (i.e., in several cells) during T-ALL development.

DNMT3A-affecting mutations, known to drive acute myeloid leukemias (AML), were observed in three adult patients in the in-house cohort and none of the pediatric T-ALLs. In fact, these three patients are classified as Early T-Cell Precursor (ETP), a T-ALL subtype that presents myeloid markers [33]. Similarly, PAT5 and PAT9, patients with mutations of ROBO2—a gene associated with progression of myelodysplastic syndrome [57] to AML and recently reported as mutated in pediatric ALL [58]—present the ETP phenotype. Clonal mutations of PHF6 are overrepresented (*χ*^2^ *p* = 0.001) in adult T-ALLs with respect to their pediatric counterparts, shared between primary and relapse samples. PHF6 is a zinc-finger transcription factor that suppresses ribosomal RNA (rRNA) transcription [32]. Loss-of-function mutations of this gene have been shown to decrease sensitivity to glucocorticoids [59], which are part of the standard first-line treatment of adult T-ALL patients. Interestingly, activating mutations of the NT5C2 gene, known to elicit resistance to mercaptopurine anti-ALL treatment in pediatric cases [26, 27], are also observed across 3 adult cases exposed to this drug (Fig. 2a), with PAT16 bearing two mutations of NT5C2 (R238G, R367Q, see Additional file 1: Table S5). In the relapse samples of two patients of the in-house cohort, we observed amplifications of ABCB1, an ATP-dependent membrane transporter known to mediate multidrug resistance in tumors [60, 61] (Additional file 2: Fig. S5). Finally, SMARCA4 mutations and deletions were also detected across adult (2) and pediatric T-ALLs, but almost exclusively in relapse malignancies, suggesting a potential role in resistance to treatment.

In summary, in 6 of the 19 adult patients of the in-house cohort, we were able to identify a candidate treatment-resistance mutation.

### The evolution of relapse adult T-ALL measured through mutations

We next asked how much do the mutational processes active in primary T-ALLs also contribute to the overall burden of mutations of relapse adult T-ALLs. The incorporation of new mutational processes, like the exposure to chemotherapies used in their treatment, could leave a mutational footprint that may be detectable in the relapse clone, as recently demonstrated in metastases of different solid tumors, and in relapsed pediatric ALL cases [37, 46].

**Fig. 3:**
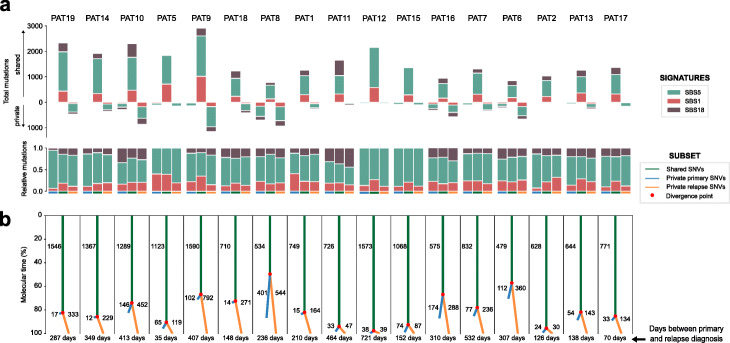
Shared and private mutations in major primary and relapse T-ALL clones. **a** Contribution of different mutational processes to the mutation burden of each T-ALL case in the adult cohort. The contribution to primary-private, relapse-private, and shared clonal mutations are indicated separately in absolute (top panel) and relative (bottom panel) terms. **b** Molecular evolution of adult T-ALL cases represented in a tree-form showing the number of shared clonal mutations (green trunk), clonal private-primary (blue branch) and clonal private-relapse (orange branch) mutations. Only signature 5 mutations are considered to build the tree (for further explanation see Additional file 2: Fig. S7). The relative length of the trunk and branches is proportional to the number of mutations in the respective group. Patients are sorted by decreasing order of age

The deconstruction of mutational signatures (representing mutational processes active during a person’s life) of primary and relapse samples of each patient reveals very similar scenarios for primary-private, shared, and relapse-private mutations (Fig. 3a). Signature 5 (SBS5), which represents a mutational process associated with hematopoietic cell division [46], contributes the vast majority (~ 80%) of mutations in these three groups. We did not detect the mutational footprint of mercaptopurine or any other chemotherapy in the relapse samples (Additional file 2: Fig. S6). This does not preclude that chemotherapy-related mutations exist below the level of detection of the sequencing technology, for example if the evolutionary bottleneck caused by the treatment has not sufficiently reduced the T-ALL population.

Since signature 5 has been described as a clock-like process [48] and this type of mutations are the main contribution to the burden of clonal mutations of both primary and relapse T-ALLs, we used them to infer a molecular time of divergence between the primary and relapse populations (Fig. 3b, Additional file 2: Fig. S7). To this end, we counted the number of primary-private, shared, and relapse-private signature 5 clonal mutations (Fig. 3b). In all cases, the branch that corresponds to relapse-private mutations is longer than that representing primary-private mutations, because the relapse clone has continued accumulating mutations longer after its divergence from the primary (eliminated as a consequence of the treatment). As expected, fewer relapse-private mutations accumulate in the cases with shorter time elapsed between the diagnosis of the primary and the emergence of relapse.

### Time of divergence of primary and relapse clones

**Fig. 4:**
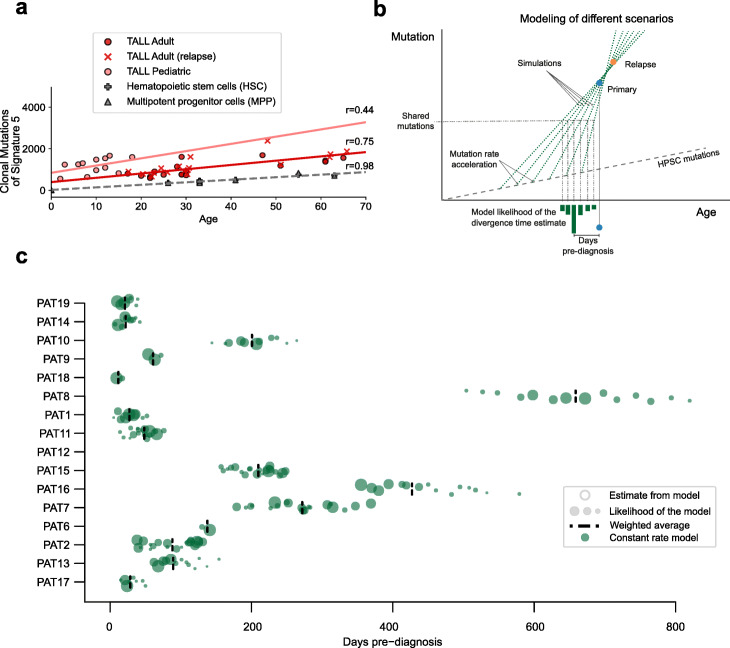
Time of divergence between major primary and relapse T-ALL clones. **a** Relationship between the mutation rate of ALL samples and the age of patients. The red line shows the regression line estimated from the data points which are the number of mutations attributed to signature 5 (red dots are primary sample and red crosses represent the relapse) of the in-house adult T-ALL cohort. In pink, the regression line estimate for the pediatric primary samples (here represented as pink dots). The gray cross and triangle correspond to the signature 5 somatic mutations from healthy tissue (MPP and HSC cells) of Osorio et al. [49]. Pearson correlation coefficient (*r*) is indicated above each of the previously mentioned regression lines. **b** Schematic representation of the different mutation rate increment models to estimate the divergence time of the leukemic (primary and relapse) cells. **c** Divergence time of the primary and relapse clone represented as days before diagnosis. The dots are the estimates computed from the models used and the size of the dots represents their likelihood (see Additional file 2: Fig. S8). The dashed line is the weighted mean of the likely model estimates (see Additional file 1: Table S7)

The number of primary-private, shared, and relapse-private signature 5 clonal mutations can also be used to estimate the precise time of the divergence of the primary and relapse clonal populations. To that end, we first needed to understand the rate of accumulation of signature 5 mutations during T-ALL development. The DNA of normal hematopoietic cells has been shown to incorporate signature 5 mutations at a rate of roughly 12 per year (Fig. 4a; Additional file 2: Fig. S7 [49]). Regressing the number of signature 5 mutations across primary and relapse T-ALLs on the age of patients in the in-house cohort in comparison with healthy hematopoietic stem cells (HSCs) yields slightly higher mutation rates and an unanticipated high (~ 400) number of mutations at the start of life of hematopoietic cells (intercept of trendline in Fig. 4a). This deviation could be explained through an acceleration in the mutation rate that occurs upon malignization of hematopoietic cells [62].

To compute the moment of time before diagnosis when this acceleration started, as well as the value of the accelerated mutation rate, we assumed that the acceleration rate is the same for the primary and relapse malignancies of a patient. We then simulated a one-time increase of the mutation rate (constant rate model) during tumor evolution and alternatively a steady increase (linear rate model) in the mutation rate for successive cell generations (Additional file 2: Fig. S8). For each patient, we assayed several trendlines of accelerated mutation rate (i.e., starting at different timepoints before diagnosis; dotted lines in Fig. 4b) approximating the observed number of signature 5 clonal mutations in the primary and relapse T-ALL clones. We computed the likelihood of each of these trends of acceleration following their accuracy to fit the observed number of mutations in the primary and relapse malignancies (Fig. 4b and Additional file 2: Fig. S8). For each trendline of accelerated mutation rate, the age of the patient at which the divergence of the two clones occurred can be computed from the number of shared mutations. The difference between this age and the age at diagnosis then yields the time elapsed between this divergence and the diagnosis of the primary T-ALL.

Upon application of this approach to each patient in the in-house cohort, we obtained a number of estimates of the number of days elapsed between the divergence of both clones and the diagnosis of the primary T-ALL, each with varying likelihood (green circles, Fig. 4c). The estimates for each patient may be summarized as their weighted (by likelihood) averages (broken lines). The time estimated for each patient was subsequently refined using the distribution of all patients (see the “Methods” section). As a result, we obtained a robust prediction of the boundaries of the most likely time elapsed between the divergence of primary and relapse clones and the diagnosis of the primary malignancy. In the majority of cases shown in the figure (13 out of 15) less than a year passed between the emergence of the relapse clone and the diagnosis of the primary disease (Additional file 1: Table S7).

### The evolution of relapse of adult T-ALLs

Both the primary and resistant populations of T blasts across the adult in-house T-ALL cohort are composed of a major clone and one or more subclones detectable through sequencing (see Additional file 3). In all the patients, including four that are refractory to treatment, the major clone in the primary and relapse leukemias differ, implying that in every case, the treatment obliterates the major clone in the primary malignancy.

To understand the effect of the therapy on the clonal architecture of adult T-ALLs, we first estimated the speed of growth of the population of T-ALL cells to determine the minimum size of the relapse population at the time of diagnosis. This growth speed may be characterized through the doubling time of the population (the time needed by a population of cells to duplicate its number). This can be computed from the number of blasts estimated by the pathologist at remission and relapse, and the amount of time elapsed between both events [37] (Additional file 2: Fig. S9a; see the “Methods” section). We computed a doubling time for the T-ALL leukemic population of 10.79 days (confidence intervals, 10.1–11.36), which is slightly longer than that recently estimated for pediatric B-ALL [37] (Additional file 2: Fig. S9b). We were then able to compute, with this doubling time, the minimum time necessary for the relapse population to achieve approximately 7 × 10^11^ cells that corresponds to a full grown leukemia [37, 63]. This minimum time to expand from a single cell upon its divergence from the primary population informs us of the likelihood that the relapse clone has arisen before the diagnosis of the primary.

**Fig. 5:**
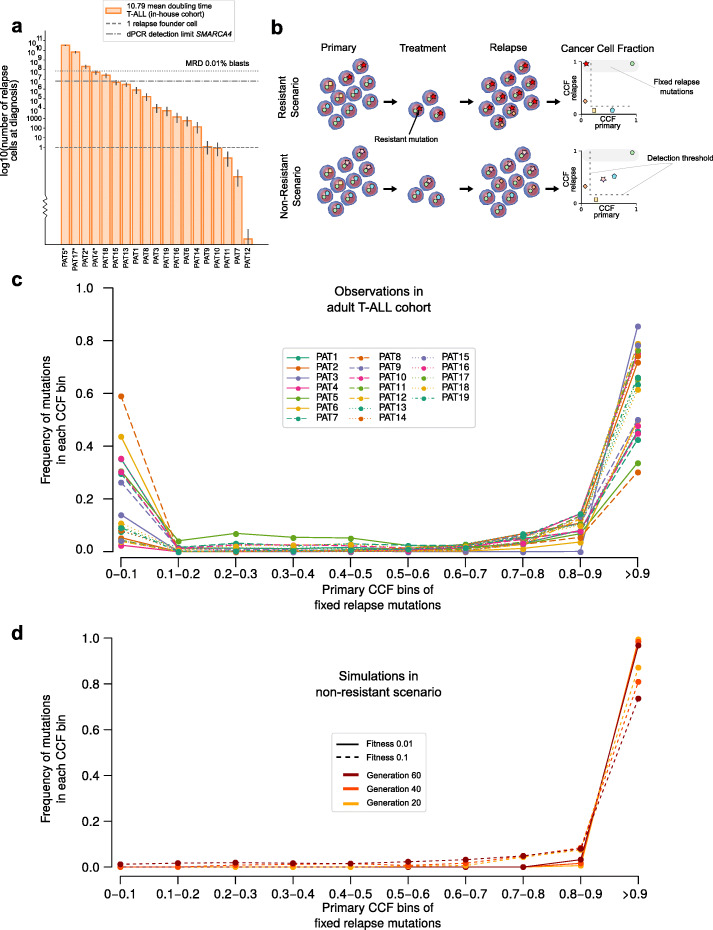
Evolution of relapse lymphoblast population. **a** Estimated size (number of cells) of the relapse population at the time of diagnosis according to the computed doubling time. Error bars represent the estimates of cell populations from the first and third quartile of the doubling time estimates, which are 10.1 and 11.36 respectively (see Additional file 2: Fig. S9). Horizontal dotted lines represent sizes corresponding to one cell and 10^8^ cells (0.01% of the population: the threshold of clinical relapse). Asterisks denote patients with estimates above the threshold of 0.01. The resolution limit of the dPCR is also represented by a horizontal line (~ 1:10,000). **b** Schematic representation of the two considered scenarios of relapse of T-ALL patients after treatment. Mutations in T-ALL cells are represented as different geometric figures. In the first scenario (resistant), one mutation in the primary T-ALL below the limit of detection of the sequencing and the digital PCR (red star) provides resistance to the treatment. All cells with this mutation survive the bottleneck posed by the treatment, and thus, this mutation and all other common to the resistant cells (hitchhikers) appear in the relapse population at CCF 1. In the second scenario (non-resistant), a group of cells with an ensemble of mutations survive the treatment. **c** Distribution (frequency) of CCF values of mutations in primary T-ALLs in the in-house cohort that are identified in their relapse counterparts as fixed (> 0.9 relapse CCF). Mutations are grouped within CCF bins. Each line represents one patient, for example, the dash brown line corresponds to PAT8, discussed in the text. **d** Distribution (frequency) of CCF values of mutations in synthetic primary T-ALL populations in evolutionary simulations following the non-resistant scenario. The dots represent mutations binned at different CCF values with the frequency that each bin represents with respect to all mutations in each synthetic relapse population. The average results of six simulation settings with different values of fitness of driver mutations and number of cell generations are presented

In three cases (PAT7, PAT11, PAT12), it is possible that the relapse clone appeared during treatment, given the estimated doubling time. In two more (PAT9 and PAT10), it is not completely clear whether there was enough time between the start of treatment and relapse to allow the emergence of a new clone. In all other cases, the relapse clone was most likely already present at the time of diagnosis and represented by more than one cell (Fig. 5a). Indeed, for fourteen patients in the cohort, the size of the relapse clone at the time of diagnosis of the primary malignancy probably comprises more than 100 out of the 7 × 10^11^ leukemia cells. (Note that this calculation is independent from the time elapsed between divergence of the primary and relapse clones and the diagnosis computed previously.) PAT2, PAT4, PAT5, and PAT17, with more than 0.01% minimal residual disease during treatment, show estimates of the relapse clone at the time of diagnosis which are, as expected, above 1 in 10,000 blasts. We then asked whether the relapse clone could be detected in the primary sample of ALL cases by a method with a lower limit of detection than Next Generation Sequencing technologies. Thus, we aimed to detect two non-synonymous SMARCA4 mutations (G1162S and T786I) that are private of the relapse sample of two patients in the corresponding primary samples of these patients (PAT8 and PAT14). With a limit of detection of around one in 1000 cells, a digital PCR was unable to detect this mutation in the primary sample of either patient (Fig. 5a and Additional file 2: Fig. S10a,b). The fraction of cells of the relapse clone estimated to be in the primary sample of these two patients is below this limit of detection. These results thus provide further support to the estimation of the doubling time and the size of the relapse clone in the primary samples derived from it.

Although we were able to pinpoint known or putative resistance mutations in several cases, we asked whether other cases of relapse could be explained by a failure of the treatment to kill a subset of the leukemic cells independent of any genetic mechanism [28, 58]. To answer this question, we modeled the emergence of the relapse clone following both a resistant and a non-resistant (not driven by a genetic mutation) scenario (Fig. 5b). First, a population of tumor cells with driver and passenger mutations was simulated. Then, to model the first scenario, a group of cells sharing one passenger subclonal mutation (the resistance mutation) were selected as survivors of the treatment and were expanded again for 20, 40, or 60 generations (40 generations correspond roughly to the observed times elapsed between primary and relapse diagnoses for the cohort; Additional file 2: Fig. S11). To simulate the second scenario, a group of cells with the same size as in the first case (but selected randomly and sharing no particular subclonal mutation) was selected and expanded for the same number of generations. We then compared the change in clonal composition—change of cancer cell fraction (CCF) of mutations in primary and relapse—obtained for both simulated scenarios with the distribution of CCF in the primary samples of mutations fixed in the relapse samples for all patients, represented in Fig. 5c. For example, of all mutations fixed in the relapse ALL of PAT8 (dashed brown line), approximately 59% were present at CCF 0–0.1% in the primary. In other words, in the primary sample, they appeared below the limit of detection of the sequencing and thus correspond to the red star mutations in the toy diagrams in Fig. 5b. On the other hand, 30% of the PAT8 fixed mutations were detected in the primary ALL at CCF between 0.9 and 1, with the remaining mutations at intermediate CCF bins. All patients in the cohort yield similar bimodal distributions.

Only in the results of the simulation of the resistant scenario do we observe a distribution of CCF of the mutations in the primary sample that resembles that of the patients in the in-house cohort (Additional file 2: Fig. S10). By contrast, in the results of the simulations of the non-resistant scenario, no mutations undetectable in the primary leukemia (CCF in the 0–0.1 decile) become fixed in the relapse (Fig. 5d). This holds if the simulations are run between 20 and 60 generations, and even if a much higher (unrealistic) fitness is assigned to driver mutations. These results suggest that the non-resistant scenario of evolution under treatment is not feasible given the time elapsed between primary and relapse.

In summary, in 14 cases in the cohort, the relapse population is most likely already present before the start of the treatment. Moreover, all relapse cases fit the model of genetic resistance—due to one genetic event common to all cells in this relapse population—although we are only able to identify the responsible mutation in a few of them.

## Discussion

Advancing our knowledge on how tumors respond to therapies and which of their features determine their relapse after treatment is key to improving clinical oncology practice. Here, we studied the genomic features and the clonal composition of nineteen adult T-ALL cases at diagnosis and after relapse to understand their evolution and identify commonalities that may predict their likelihood to respond to current therapeutic approaches.

Our results suggest that for most adult T-ALL patients, the population of leukemia cells that dominates the relapse is already present at the moment of diagnosis, that is before the start of the treatment, and comprises more than one but fewer than 10^8^ blasts. One evidence that supports this notion comes from the fact that, in most cases, the span of time between the diagnosis and the emergence of relapse is not enough (given the doubling time estimated from the cohort) to explain the repopulation of a full leukemic population starting from a single cell. This contrasts with the results reported recently for a pediatric cohort, in which some relapse cases could be explained by resistance mutations appearing during treatment [37]. This finding is relevant for the clinical practice, since early identification of such potential resistance populations in a patient’s leukemia may support making clinical decisions regarding their treatment.

We were not able to detect the mutational footprint of chemotherapies employed in the treatment of patients of this cohort, such as mercaptopurine, which has already been characterized in pediatric T-ALL cases [37]. This does not preclude that these chemotherapies indeed cause mutations in leukemic cells that progress in the relapse. Since upon treatment chemotherapy mutations will be private to each blast, if the relapse clone does not emerge from a complete clonal expansion after the start of the treatment, the variant allele frequency of these treatment mutations will not rise above the limit of detection of the sequencing. The detection in the relapse T-ALL population [37] of these treatment mutations would require that only one or few blasts survived the treatment, guaranteeing that sufficient numbers of cells in the relapse carried the same mutations to make them detectable through sequencing. The absence of treatment footprints in the relapse is therefore another evidence that the relapse population at the time of treatment already contains a large number of cells.

One intriguing result is the detection of multiple mutations affecting the NOTCH pathway in the same T-ALL case, which do not appear to be exceptions, but rather the rule. It is possible that mutations affecting different domains of NOTCH1 increase the fitness of leukemic cells more than a single mutation and provide an advantage for relapse. Further studies comparing the pattern of NOTCH1 mutations in relapsing and non-relapsing T-ALLs are needed to clarify this.

## Conclusions

All results show that, in the T-ALL patients of this cohort, the relapse is driven by genetic mutations that appear in the population of blasts several months before diagnosis, giving rise to a resistant subclone of up to several million cells at the beginning of treatment. Upon treatment thus, this subclone comes to dominate the T-ALL population at relapse.

## Methods

### In-house cohort selection and samples collection

Samples from adults (≥ 18 years old) with T cell acute lymphoblastic leukemia were collected in the course of 15 years under therapy protocols (LAL-07OLD, ALL-HR-03, LAL-AR-2011) as part of the PETHEMA (Programa Español de Tratamientos en Hematología) trials (with the exception of patient 16). Patients have signed the corresponding consents of the protocols. Cohort clinical data is specified in Additional file 2: Fig. S3 and Additional file 1: Table S1. There are three collected samples per patient: one taken at diagnosis (primary), a second one when the percentage of lymphoblasts is reduced during treatment (remission), and a final sample when the leukemia reappears after some months (relapse).

### Whole genome sequencing

The short-insert paired-end libraries for the whole genome sequencing were prepared with KAPA HyperPrep kit (Roche Kapa Biosystems) with some modifications. In short, in function of available material 0.1 to 1.0 microgram of genomic DNA was sheared on a Covaris™ LE220-Plus (Covaris). The fragmented DNA was further size-selected for the fragment size of 220–550 bp with Agencourt AMPure XP beads (Agencourt, Beckman Coulter). The size selected genomic DNA fragments were end-repaired, adenylated, and ligated to Illumina platform compatible adaptors with Unique Dual matched indexes or Unique Dual indexes with unique molecular identifiers (Integrated DNA Technologies). The libraries were quality controlled on an Agilent 2100 Bioanalyzer with the DNA 7500 assay for size and the concentration was estimated using quantitative PCR with the KAPA Library Quantification Kit Illumina® Platforms (Roche Kapa Biosystems). To obtain sufficient amount of libraries for sequencing, it was necessary for the low input libraries (0.1–0.2 μg) to amplify the ligation product with 5 PCR cycles using 2x KAPA-HiFi HS Ready Mix and 10X KAPA primer mix (Roche Kapa Biosystems).

The libraries were sequenced on HiSeq 4000 or NovaSeq 6000 (Illumina) with a paired-end read length of 2 × 151 bp. Image analysis, base calling, and quality scoring of the run were processed using the manufacturer’s software Real Time Analysis (HiSeq 4000 RTA 2.7.7 or NovaSeq 6000 RTA 3.3.3).

### Analysis of ALL cohorts in the public domain

We downloaded public whole-genome and whole-exome sequencing data from EGA and dbGap. We included samples from St. Jude Children’s Research Hospital associated with EGAD00001001052 and EGAD00001001432 EGA accession codes. We have used only samples of which we could recover clinical information from the associated publications [5, 8, 10, 38, 39]. We downloaded the DNA sequencing data of Oshima et al., 2016 [30] from dbGap under the accession code phs001072.v1.p1. The information of the cohorts with the clinical information gathered for each sample is summarized in Additional file 1: Table S2.

For some of the samples, we could not find information regarding the sex of the patient. In those cases we inferred it from the normal sample BAM of each patient. For that, we applied the following reasoning: (1) we determined that the patient is a female if the average coverage of chromosome X is greater than the minimum average coverages of the autosomal chromosomes and (2) the mean coverage of chromosome Y is 10 times smaller than the average coverage of the autosomal chromosomes of the sample.

All the samples in Additional file 1: Table S2 have been analyzed with the same pipeline (for detailed information see the following section: “Alignment and variant calling”). However, in order to compare the T-ALL Adult cohort with other T-ALL cohorts with pre- and post-treatment samples, we added the mutations reported in the supplementary materials in Li et al. [37] only in Fig. 2a and b.

### Alignment and variant calling

#### Alignment, SNV, small InDels

We performed the alignment and calling of mutations (SNVs and small InDels) using Sarek pipeline v2.2.1 [64]. This workflow performs the alignment from raw FASTQ applying the steps referred to as “best practices” according to GATK. We converted the downloaded BAMs from public repositories to FASTQ with biobambam v2.0.72 and used them as input for the pipeline. We used the Strelka caller implemented in Sarek to generate mutation calls. Only the T-ALL adult cohort was aligned with GEM-mapper v3.6 by the CNAG but the calls were done with Strelka. The mutation calls were performed using primary and relapse as tumor samples and the remission as “normal” sample. Variants have been annotated with VEP v.92 run locally with the canonical flag and using gnomAD r2.0.1 to get population frequencies of the potential polymorphisms.

#### CNV

We have used FACETS v0.5.6 [65] to call copy number changes in WGS and WES samples. Following FACETS documentation, we first created its input with snp-pileup which imputed common SNPs and made the reference and alternative read counts at nucleotide resolution. We have run snp-pileup with the recommended parameters except for the --min-read-counts that was set to 10,0. We run FACETS for WES as mentioned in the documentation but setting preProcSample function parameters to cval = 15, ndepth = 5, snp.nbhd = 500 and procSample function parameters to cval = 80, min.nhet = 20. Similarly, we run FACETS for the WGS data as preProcSample (snp.nbhd = 5000, ndepth = 5, cval = 75) and procSample (cval = 800, min.nhet = 25).

#### SV

We ran Delly v0.7.9 [66] to detect duplications, inversions, and translocations. First we ran the *call* function and then the *filter* function of Delly for each one of the alterations mentioned. The map-quality parameter of the call function was set to 20 and we also passed a file provided in the github of Delly with regions to exclude through the --exclude argument. The filter function was run with the following parameters: --filter somatic --minsize 0 (expect for duplications which was set to 100) --qual-tra 0.75 --altaf 0.1.

### Filtering steps

#### SNVs and InDels

From the VCF output from Strelka, we retained the calls labeled as PASS and DP from the FILTER column. We recovered the shared mutations between primary and relapse that are not PASS or DP but are present in the original VCF. This was not possible for patients with only paired samples (primary and remission) in some cohorts. In addition, we checked for miss-called DNVs (dinucleotide variants) by inspecting consecutive SNV positions with Samtools v1.4.1 and changed the reference and alternative if needed. Once the variants were annotated with VEP, we took the variants in the canonical transcript. In case of more than one consequence type predicted for the same variant, we took the most damaging (more impact) one according to VEP. We also filtered out mutations with population frequency greater than 0.01 according to the gnomADg_AF column added. Finally, we discarded low coverage variants as the ones with a total depth of 5 reads. Further details regarding filters applied to called SNVs are provided in Additional file 3.

#### CNV

We discarded the variants that were called with low reliability. Those are the segments reported with NAs in the cellular fraction and minor allele copy number columns of FACETS output which, to our knowledge, indicate that the region does not have sufficient numbers of heterozygous SNPs to guide good estimates (Additional file 2: Fig. S5).

#### SV

We converted the VCFs into bedpe format with bcftobedpe function from svtools v0.4.0 and kept the variants with the flag PASS in the FILTER column. We manually check recurrent SV that have not been described before in the literature by performing BLAT of the breakend points (BND) and their flanking regions in the UCSC and discarded those that were Alu regions or mappable to many parts of the genome.

### Purity and clonality estimations

We inferred the purity of the samples from the variant allele frequency (VAF) distribution of the mutations as follows. Since the overall ploidy of the samples was mostly around 2 (diploid), we computed density plots of the VAF multiplied by the CNV of each mutation as a rough proxy of the CCF and determined the purity as the maximum point. We recomputed the CCF with the inferred purity and fitted a beta binomial distribution (betabinom function from scipy v1.4.1 python package). For each mutation, we derived a probability from it and categorized them as clonal or subclonal according to a threshold of 0.01 (above or below it respectively). Exceptionally for PAT16, upon inspection of the CCF distributions in primary and relapse samples, we detected a more complex clonal structure and thus used a threshold of 0.05 for a clearer categorization of the clonality of the mutations.

### Signatures analysis

Several runs of deconstructSigs v.1.8.0 [67] were carried out depending on the context of the analysis. Firstly, following the guidelines proposed by Maura et al. [50], we have included all hematological meaningful described signatures for the fitting of primary samples (see Additional file 2: Fig. S1). From those, we selected the signatures that we believed had a substantial activity in the primary leukemias in at least one patient of the cohort analyzed and re-run deconstructSigs with them (see Fig. 1c). Secondly, we re-fitted the T-ALL adult samples with only those signatures that presented activity (SBS1, SBS5, SBS18) to better estimate their contribution in Fig. 3a. Lastly, we have fitted known-treatment signatures for the primary and relapse samples to see whether there is any contribution of those in the mutational profile of the relapse. In this case, we have included Signature 32 (SBS32) which the proposed etiology in COSMIC [68] suggests prior treatment with azathioprine. The adult T-ALL patients have not been treated directly with this compound but it is known that azathioprine is metabolized to 6-mercaptopurine which is used in the maintenance phase of received therapy (see Additional file 2: Figs. S3 and S6). Apart from SBS32, we have also included two treatment signatures recently extracted in Li et al. [37] as SBSA_new and SBSB_new. They assigned the usage of thiopurines to SBSB_new signature so that is why we have decided to include it. There is not much said about SBSA_new but since pediatric and adult ALL patients receive similar treatment we decided to give it a try in the fitting analysis. In all cases, we set the signature cutoff parameter of deconstructSigs to 0.1.

### Clustering of driver genes of ALL subtypes

The distances computed to build the dendrogram on Fig. 1d were based on Jensen-Shannon divergence measures between the distributions of the number of patients per mutated gene of each cohort. We only took into account genes with mutations in at least three patients.

### Dimensionality reduction

We used a Uniform Manifold Approximation and Projection (UMAP) implemented in the python package umap-learn v0.3.10 to simplify the mutational profiles (96 dimensions that represent each trinucleotide channel) into two dimensions with the size of the local neighborhood (n_neighbors) to 20 and minimal distance (min_dist) of 0.2.

### Identification of ALL driver variants

#### Driver gene discovery

We have run the IntOGen pipeline [69] for SNVs and small InDels (https://www.intogen.org/search) locally for each of the defined cohorts (see above). For each one of the outputs, we have proceeded as follows. First, we have discarded all genes in Tier 3 and 4 that are not in the Cancer Gene Census (CGC) [70]. Second, we have discarded all genes in all tiers that have been defined as potential artifacts (see this list of genes in https://bitbucket.org/intogen/intogen-plus/src/master/extra/data/artifacts.json). Third, we have manually inspected the remaining genes and defined a list of potential false positives (FP). From this list of suspicious genes, we have discarded those not present in the CancerMine. With the rest of the FP candidates that were present in the CancerMine, we have decided their level of credibility as driver genes of leukemia according to the publications reported. Apart from that, we have also manually searched in PubMed for any other missed relation by CancerMine of the gene and hematopoietic neoplasms (see Additional file 1: Table S3).

#### Literature lists of cancer genes of ALL

We have defined 3 lists of known driver genes in ALL:

- Genes with SNVs/InDels mutations
- Genes affected by CNV
- Genes affected by SV that are known to drive ALL

The genes and their sources to build these lists are listed in Additional file 1: Tables S4.a,b,c respectively.

#### Annotation of alterations

For SNVs and InDels, we have defined as potential driver all the mutations with a predicted protein affecting consequence type (in the canonical transcript) according to VEP (transcript_ablation, splice_acceptor_variant, splice_donor_variant, stop_gained, frameshift_variant, stop_lost, start_lost, transcript_amplification, inframe_insertion, inframe_deletion, missense_variant, protein_altering_variant, splice_region_variant, incomplete_terminal_codon_variant, start_retained_variant, stop_retained_variant) in a cancer gene from the list defined as the combination of the results from the Driver Gene Discovery and the curated literature list of SNVs and InDels. Results from that are summarized in Fig. 1d, Additional file 2: Fig. S2, and Additional file 1: Table S5.

For CNV and SV, we have flagged the alterations we have found as “known driver” (contained in the curated literature lists respectively) or with “alteration in gene of interest” if it affects any cancer gene related to leukemia of all the lists. In the case of CNV affecting genes of interest, we consider as candidate drivers those oncogenes that are fully amplified and tumor suppressors affected by any deletion. Results are reported with the annotated “classic” Giemsa cytobands by mapping where the BND genomic coordinates fall within them (see Additional file 1: Table S6 a and b).

We have also annotated the genes affected grouping them by some meaningful information such as their protein family, biological process, or pathway (see Additional file 2: Figs. S2, S4 and Additional file 1: Table S4). We created those groups with information from the sources in Additional file 1: Table S4.

### Estimations of divergence time

Considering the differences between the mutational burden of T-ALL samples compared with the expected number of mutations of healthy hematopoietic cells seems clear that some acceleration on the mutation rate has occurred (Fig. 4a). Additionally, the regression between age and signature 5 of healthy cells and T-ALL show close slope (12.21 ∓ 1.24 vs 20.61 ∓ 6.58, see Fig. 4a and Additional file 2: Fig. S7) but a much higher intercept (22.35 ∓ 45.53 vs 397.4 ∓ 251.81, see Fig. 4a and Additional file 2: Fig. S7). We hypothesize these similarities on slope and differences on intersect can be explained by a late-stage acceleration during tumorigenesis that affects in a similar way the different T-ALL samples.

Based on this hypothesis of tumorigenesis acceleration of signature 5, we have built 2 different models which represent the upper and lower boundary of the estimations: (I) the change of mutation rate is a one-time, discontinuous event, shared between primary and relapse, and (II) the change on the mutation rate grows linearly during all lifetime of the tumor. In both scenarios, the mutation rate can only increase and both primary and relapse clones are under the same mutational process. In terms of divergence time, the constant model is the most conservative showing the earliest times of divergence between clones, while the linear model is the one generating larger divergences times. The rest of the models based on N acceleration steps will generate estimates within the previous described.

We established 120 different timepoints *t*_n_ evenly spaced along the 10-year period immediately preceding diagnosis: we refer to them as “acceleration times,” since they are bound to represent the time-points when the mutation rate first deviates from neutral, clock-like behavior. For each acceleration time, we first computed a function assigning a plausible mutation rate for each time point, consistently with either the constant or linear model. To this end, we fitted the mutation curve to go through the average number of mutations of primary and relapse *N*(t*) at the middle timepoint t* between these two events. More specifically, the following conditions must hold:

$$ \mathrm{Constant}:N\left({t}^{\ast}\right)=N\left({t}_n\right)+\mu \cdotp \left({t}^{\ast }-{t}_n\right) $$

$$ \mathrm{Linear}:N\left({t}^{\ast}\right)=N\left({t}_n\right)\cdotp {\left(1+r\right)}^{t^{\ast }-{t}_n} $$

where the values of *μ* and r have to be determined, depending on the model used. Now we did 100 stochastic simulations of the mutation curve by randomly sampling 0 or 1 mutations from a beta binomial distribution with a 1-day granularity, only in cases the mutation rate per day exceeds one a smaller granularity has been used. Thus, mean parameter *μ*(*t*) may change with time (linear model) while correlation parameter *ρ* = 0.0002, estimated with the dispersion observed on healthy hematopoietic stem cells described on Osorio et al. [49], remains constant. Therefore, the number of mutations simulated at time *t* is defined recursively as:

$$ N\left({t}_m\right)\sim N\left({t}_{m-1}\right)+ BetaBinom\left(\mu \left({t}_m\right),\rho, 1\right) $$

where *μ*(*t*_*m*_) is either *μ* (constant model) or *log*(1 + *r*) · *N*(*t*_*m* − 1_) (linear model). As the 100 stochastic curves generated for each hypothesis (determined by the acceleration time and mutation rate model) cut the time levels at primary and relapse, they cast a distribution of the possible number of mutations about the observed that yields a likelihood that the hypothesis explains well the observed number of mutations at primary and relapse. Thus, each combination of acceleration time and mutation rate model has an associated prior likelihood. We calculated the Bayes posterior distribution using the combinations of parameters with a higher success (likelihood) on the cohort which is then used to select the most plausible models underlying the observation, then provide a plausible set of divergence times weighted by the likelihood. In order to avoid the deviation of the divergence time estimation due to a long tail of low likelihood simulations, only the more likely scenarios have been selected (10% percentile).

### Doubling time and lymphoblast population estimates

The doubling time of the T cell lymphoblast population was estimated following a similar approach as in Li et al. [37]. We assumed that blast cell growth is consistent with a logistic model, i.e., the population fraction represented by the T-lymphoblast population as a function of time *t* fits a logistic function of the form:

$$ \sigma \left(t,a\right)={\left(1+{e}^{- at}\right)}^{-1} $$

where *a* is the parameter of the logistic model and *t* is assumed to be given in standard time units such that the T-lymphoblast subpopulation reaches 50% of the total population at time *t* = 0.

Assuming the parameter *a* is known, the doubling time is given by the following expression:

$$ {T}_D=\mathit{\log}(2)/a $$

Therefore, the doubling time estimate resorts to fitting a logistic model to our data, i.e., provide an estimate for the parameter *a*.

Our approach intends to provide an estimate of *a* that corrects for the likely inconsistencies between time annotations provided in the patients’ data. We make the general assumption that some error *Δt*_*i*_ has been introduced for each patient *P*_*i*_ when associating a standard time to the T-lymphoblast population measurements—mainly due to the difficulty to estimate the initial time for paired data points with a low initial T-lymphoblast population fraction. A standard goodness-of-fit criterion for logistic models is given by the cross-entropy loss:

$$ C\left(y,\hat{y}\right)=-\frac{1}{n}{\sum}_{i=1}^n{y}_i\mathit{\log}{\hat{y}}_i+\left(1-{y}_i\right)\mathit{\log}\left(1-{\hat{y}}_i\right) $$

where *y* and $\hat{y}$ are the observed (resp. predicted) data samples.

Our approach intends to simultaneously estimate the errors *Δt*_*i*_ and the parameter a by minimizing the following cross-entropy loss:

$$ L\left(a,\varDelta {t}_1,\dots, \varDelta {t}_n\right)=-\left({\sum}_{i=1}^nC\left({y}_{i,0};{t}_{i,0};\varDelta {t}_i\right)+C\left({y}_{i,1};{t}_{i,1};\varDelta {t}_i\right)\right) $$

where *C*(*y*; *t*; *Δt*) = *ylogσ*(*t* − *Δt*, *a*) + (1 − *y*) *log* (1 − *σ*(*t* − *Δt*, *a*))

where for each patient *P*_*i*_ the values *y*_*i*, 0_ and *y*_*i*, 1_ are the initial (resp. final) population fractions and the values *t*_*i*, 0_ and *t*_*i*, 1_ are the initial (resp. final) times.

Minimization of the cross-entropy *L* was implemented in Python with the function “minimize” of the scipy.optimize module. For a more robust minimization, we ran it several times with different randomly generated initial values (see Additional file 2: Fig. S9).

Upon estimation of the doubling time *T*_D_, we proceed to compute the number of cells *N*_d_ at the time of diagnosis as a function of the time *Δt* elapsed between diagnosis and relapse:

$$ {N}_{\mathrm{d}}={N}_{\mathrm{B}}\cdotp f\cdotp {2}^{-\varDelta t/{T}_{\mathrm{D}}} $$

where *N*_B_ is an estimate of the total number of bone marrow cells in adults (~ 7.5 × 10^11^ cells according to [37, 63]) and *f* is the frequency of lymphoblasts of the biopsy.

### Digital PCR analysis of *SMARCA4* mutations

The dPCR analysis was performed on a QuantStudio 3D dPCR System using the manufacturer’s procedure and reagents (ThermoFisher Scientific). Data analysis and chip quality were assessed using the QuantStudio 3D Analysis Suite software online.

### Simulations of relapse scenarios

In order to understand how likely our observations at primary and relapse can be obtained under a non-therapy selective scenario, we have performed several simulations using a Wright-Fisher model (https://github.com/gerstung-lab/clonex).

Firstly, we have established a set of parameters based on our observations of primary samples using a mutation rate of 10^−8^ and a total number of driver and passenger positions of 100 (0.01 fitness effect) and 150,000 respectively on a population of 10^6^ cells. As a result, after 5000 generations, the population has fixed a number of driver mutations ranging from 3 to 8 (mean 5.2) and 122 to 753 (mean 505.8) passengers.

Secondly, from the primary population we randomly removed between 9 × 10^4^ and 10^6^ cells to simulate a bottleneck effect. The resulting population has grown for 20, 40, and 60 generations which covers our estimations about the observed dataset (10% CI 10.83–37.89 generations).

Finally, we have compared the VAF distribution at primary of those variants with a VAF at relapse higher than 90%, considered as fixed mutations, between the observed and simulated non-resistant scenario.

Due to the lack of fixation of low VAF variants in our simulations, two additional scenarios were performed under the previously described strategy: (I) A non-resistant simulation increasing the fitness up to 0.1 (considered as high fitness, [71]) to allow for faster fixation rates and (II) a resistant scenario where the bottleneck consists of the selection of all cells sharing a low population frequency passenger mutation, defined as resistant mutation.

## Supplementary information

**Additional file 1.** Additional tables. This file contains the supplementary tables referenced in the main text. Table S1 contains clinical information on the adult T-ALL cohort. Table S2 contains clinical information of the public pediatric cohorts. Table S3 contains the detected cancer genes by IntOGen. Table S4 contains the lists of ALL cancer genes of interest found in the literature separated in 3 subtables according to the type of alterations: SNVs and InDels (Table S4.a), CNV (Table S4.b), SV (Table S4.c). Table S5 contains the mutations (SNVs and InDels) that we consider as candidate drivers. Table S6 has the candidate driver CNVs (Table S6.a) and SVs (Table S6.b) of the cohorts analyzed. Table S7 has the time of divergence estimates between primary and relapse estimated as days pre-diagnosis of each patient.

**Additional file 2.** Additional figures. This file presents all supplementary figures referenced in the main text.

**Additional file 3.** Additional methods. Some of the filtering steps have been extended for clarification in this file.

**Additional file 4.** Review history.

## Data Availability

The raw data of the genomic sequencing of the 45 samples (primary-remission-relapse) of the patients of the in-house cohort is deposited in the EGA repository (accession code EGAS00001004750 [72]). To facilitate reproducibility, the code of the analysis is available here: https://github.com/bbglab/evolution_TALL_adults under Apache Software License 2.0 (doi:10.5281/zenodo.4120326 [73];). Raw sequencing data of public datasets produced by St. Jude Children’s Research Hospital-Washington University Pediatric Cancer Genome Project (see Table 1) was obtained from the EGA repository (accession codes EGAD00001001052 and EGAD00001001432; some BAMS corresponding to published projects somewhere else [5, 6, 8, 10, 14, 38]). Raw sequencing data of patients included in the study by Oshima et al. [30] (Table 1) was obtained from dbGap (phs001072.v1.p1). The somatic mutations identified in the patients included in the study by Li et al. [37] were obtained from the Supplementary Data of the original paper.
