# Supplementary material for: The evolution of relapse of adult T cell acute lymphoblastic leukemia
Source: Genome Biol. 2020 Nov 23;21:284. doi: 10.1186/s13059-020-02192-z (PMC7682094; doi:10.1186/s13059-020-02192-z)
Supplement: Supplementary file 2 — Additional file 2. Additional figures. This file presents all supplementary figures referenced in the main text. [file 13059_2020_2192_MOESM2_ESM.pdf]

Fig. S1

a

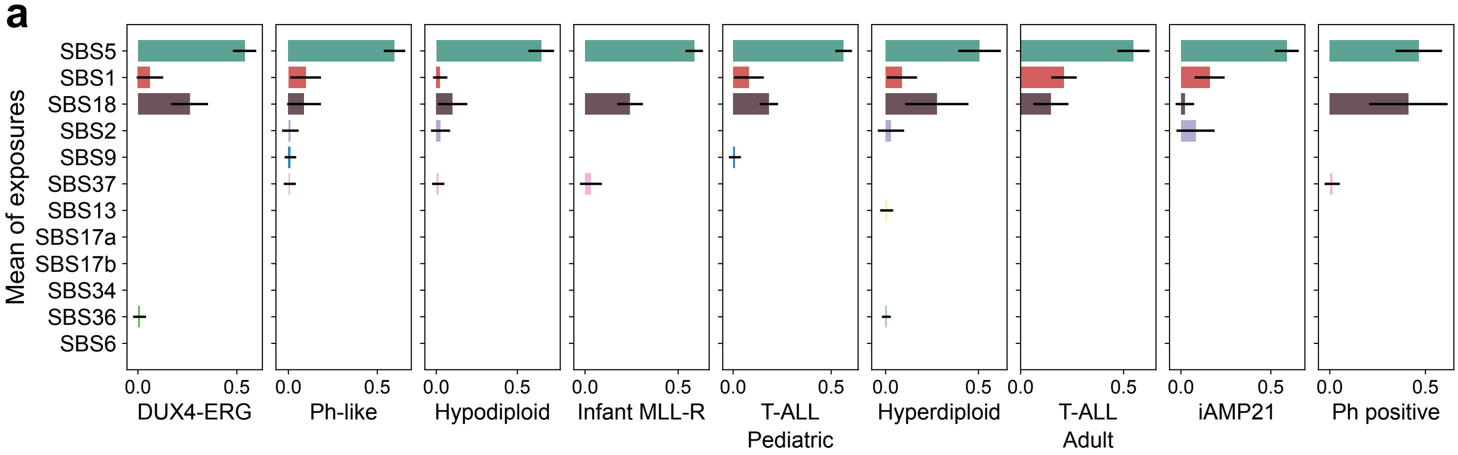
Mean of exposures
SBS5
SBS1
SBS18
SBS2
SBS9
SBS37
SBS13
SBS17a
SBS17b
SBS34
SBS36
SBS6
0.0
0.5
DUX4-ERG
Ph-like
Hypodiploid
Infant MLL-R
T-ALL Pediatric
Hyperdiploid
T-ALL Adult
iAMP21
Ph positive

Fig. S1. Probing for active mutational processes across ALL cohorts

Mutational processes active across primary ALL cohorts, represented by their mean (and standard deviation) contribution of the mutation burden of each cohort. The list of signatures to fit was determined by their activity in any hematopoietic cancer according to COSMIC (see Supp. Methods). This linear fitting (Methods), was used to select the subset of mutational processes active in ALL tumors of the studied cohorts which are shown in the main Figures.

Fig. S2

a

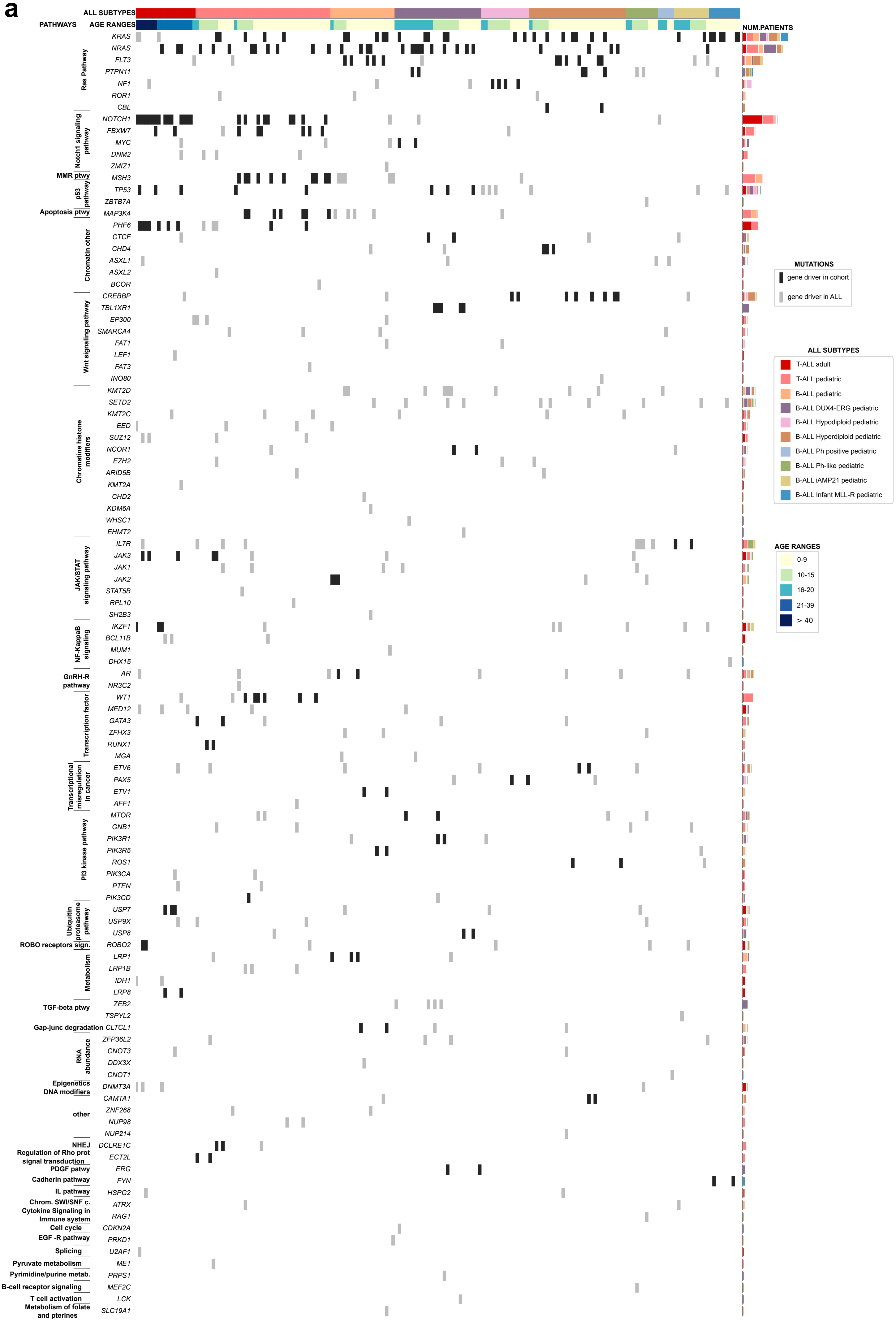

ALL SUBTYPES
PATHWAYS
AGE RANGES
NUM.PATIENTS
Ras Pathway
Notch1 signaling pathway
MMR ptwy
p53 pathway
Apoptosis ptwy
Chromatin other
Wnt signaling pathway
Chromatine histone modifiers
JAK/STAT signaling pathway
NF-KappaB signaling
GnRH-R pathway
Transcription factor
Transcriptional misregulation in cancer
PI3 kinase pathway
Ubiquitin proteasome pathway
ROBO receptors sign.
Metabolism
TGF-beta ptwy
Gap-junc degradation
RNA abundance
Epigenetics DNA modifiers
other
NHEJ
Regulation of Rho prot signal transduction
PDGF patwy
Cadherin pathway
IL pathway
Chrom. SWI/SNF c.
Cytokine Signaling in Immune system
Cell cycle
EGF -R pathway
Splicing
Pyruvate metabolism
Pyrimidine/purine metab.
B-cell receptor signaling
T cell activation
Metabolism of folate and pterines
KRAS
NRAS
FLT3
PTPN11
NF1
ROR1
CBL
NOTCH1
FBXW7
MYC
DNM2
ZMIZ1
MSH3
TP53
ZBTB7A
MAP3K4
PHF6
CTCF
CHD4
ASXL1
ASXL2
BCOR
CREBBP
TBL1XR1
EP300
SMARCA4
FAT1
LEF1
FAT3
INO80
KMT2D
SETD2
KMT2C
EED
SUZ12
NCOR1
EZH2
ARID5B
KMT2A
CHD2
KDM6A
WHSC1
EHMT2
IL7R
JAK3
JAK1
JAK2
STAT5B
RPL10
SH2B3
IKZF1
BCL11B
MUM1
DHX15
AR
NR3C2
WT1
MED12
GATA3
ZFHX3
RUNX1
MGA
ETV6
PAX5
ETV1
AFF1
MTOR
GNB1
PIK3R1
PIK3R5
ROS1
PIK3CA
PTEN
PIK3CD
USP7
USP9X
USP8
ROBO2
LRP1
LRP1B
IDH1
LRP8
ZEB2
TSPYL2
CLTCL1
ZFP36L2
CNOT3
DDX3X
CNOT1
DNMT3A
CAMTA1
ZNF268
NUP98
NUP214
DCLRE1C
ECT2L
ERG
FYN
HSPG2
ATRX
RAG1
CDKN2A
PRKD1
U2AF1
ME1
PRPS1
MEF2C
LCK
SLC19A1
MUTATIONS
gene driver in cohort
gene driver in ALL
ALL SUBTYPES
T-ALL adult
T-ALL pediatric
B-ALL pediatric
B-ALL DUX4-ERG pediatric
B-ALL Hypodiploid pediatric
B-ALL Hyperdiploid pediatric
B-ALL Ph positive pediatric
B-ALL Ph-like pediatric
B-ALL iAMP21 pediatric
B-ALL Infant MLL-R pediatric
AGE RANGES
0-9
10-15
16-20
21-39
> 40

Fig. S2. Mutations in driver genes in primary ALLs

Rows are driver genes in ALL (collected from the literature or identified across these cohorts using IntOGen; see Supp. Methods) grouped by protein family, biological process or pathway. Columns are ALL samples grouped by cohort, and sorted by age. Each full rectangular cell represents a protein-affecting mutation in a driver gene annotated from the literature (grey) or directly detected as driver in that cohort through the IntOGen pipeline (black). The bars on the left represent the total number of patients in each ALL cohort with mutations of the gene.

Fig. S3

a

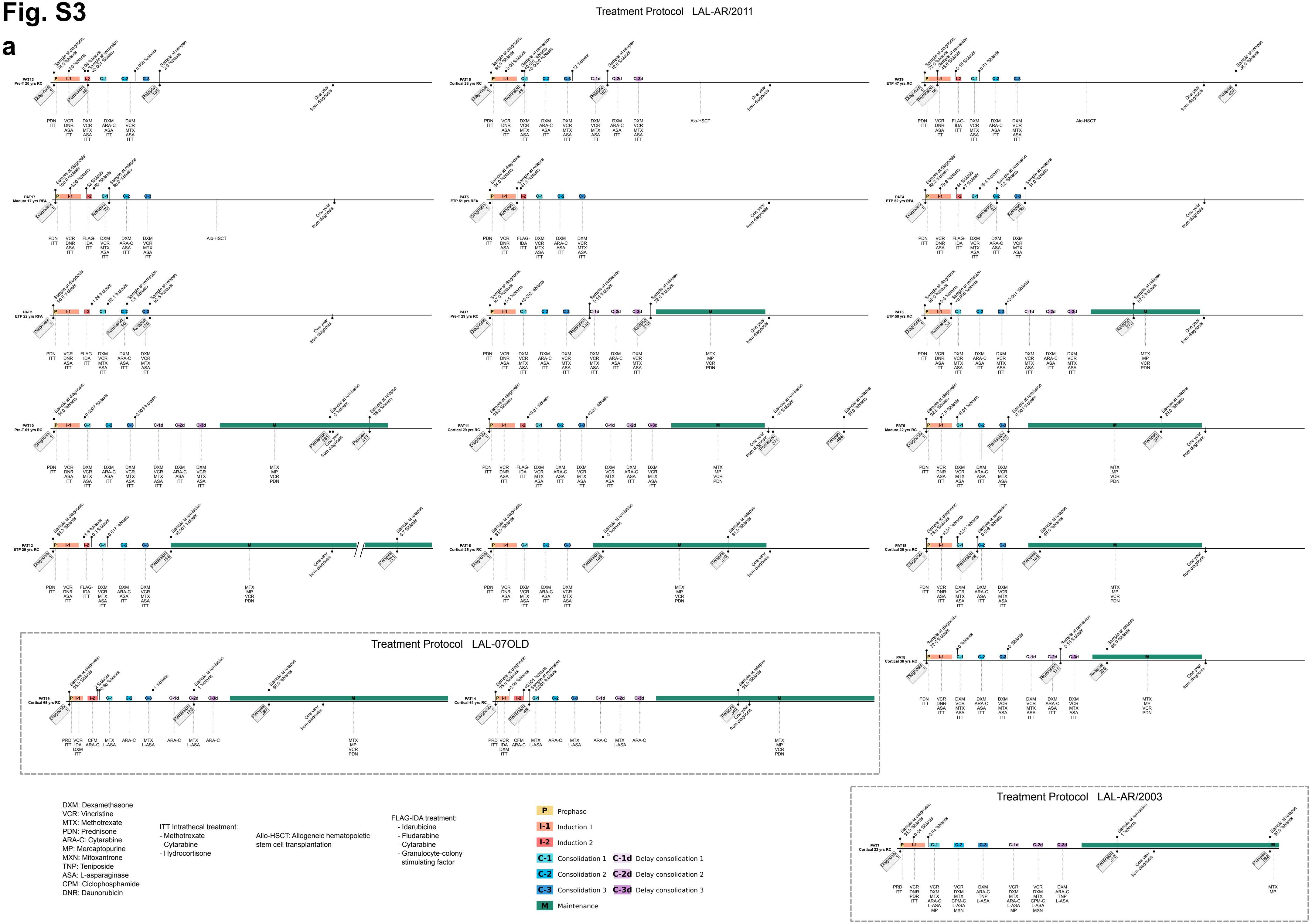

Treatment Protocol LAL-AR/2011
Treatment Protocol LAL-07OLD
Treatment Protocol LAL-AR/2003
DXM: Dexamethasone
VCR: Vincristine
MTX: Methotrexate
PDN: Prednisone
ARA-C: Cytarabine
MP: Mercaptopurine
MXN: Mitoxantrone
TNP: Teniposide
ASA: L-asparaginase
CPM: Ciclophosphamide
DNR: Daunorubicin
ITT Intrathecal treatment:
- Methotrexate
- Cytarabine
- Hydrocortisone
Allo-HSCT: Allogeneic hematopoietic stem cell transplantation
FLAG-IDA treatment:
- Idarubicine
- Fludarabine
- Cytarabine
- Granulocyte-colony stimulating factor
P Prephase
I-1 Induction 1
I-2 Induction 2
C-1 Consolidation 1
C-2 Consolidation 2
C-3 Consolidation 3
C-1d Delay consolidation 1
C-2d Delay consolidation 2
C-3d Delay consolidation 3
M Maintenance

Fig. S3. Clinical course of patients in the in-house T-ALL cohort.

Each figure represents the clinical course of a patient. Day 1 represents the sample taken at diagnosis and the total extension of the line comprises one and a half years. Lymphoblast counts at regular checks and sample extractions performed at the hospital are represented above the line. Colored boxes represent treatment cycles according to clinical protocols (see Supp. Methods), and rectangular grey labels below correspond to sample extraction timepoints. The width of the treatment box is approximately scaled to the time that corresponds to protocols guidelines. Treatments received appear below the timelines. PAT16 most likely received treatment based on protocol LAL-AR/2011, however, this information has never been confirmed.

Fig. S4

a

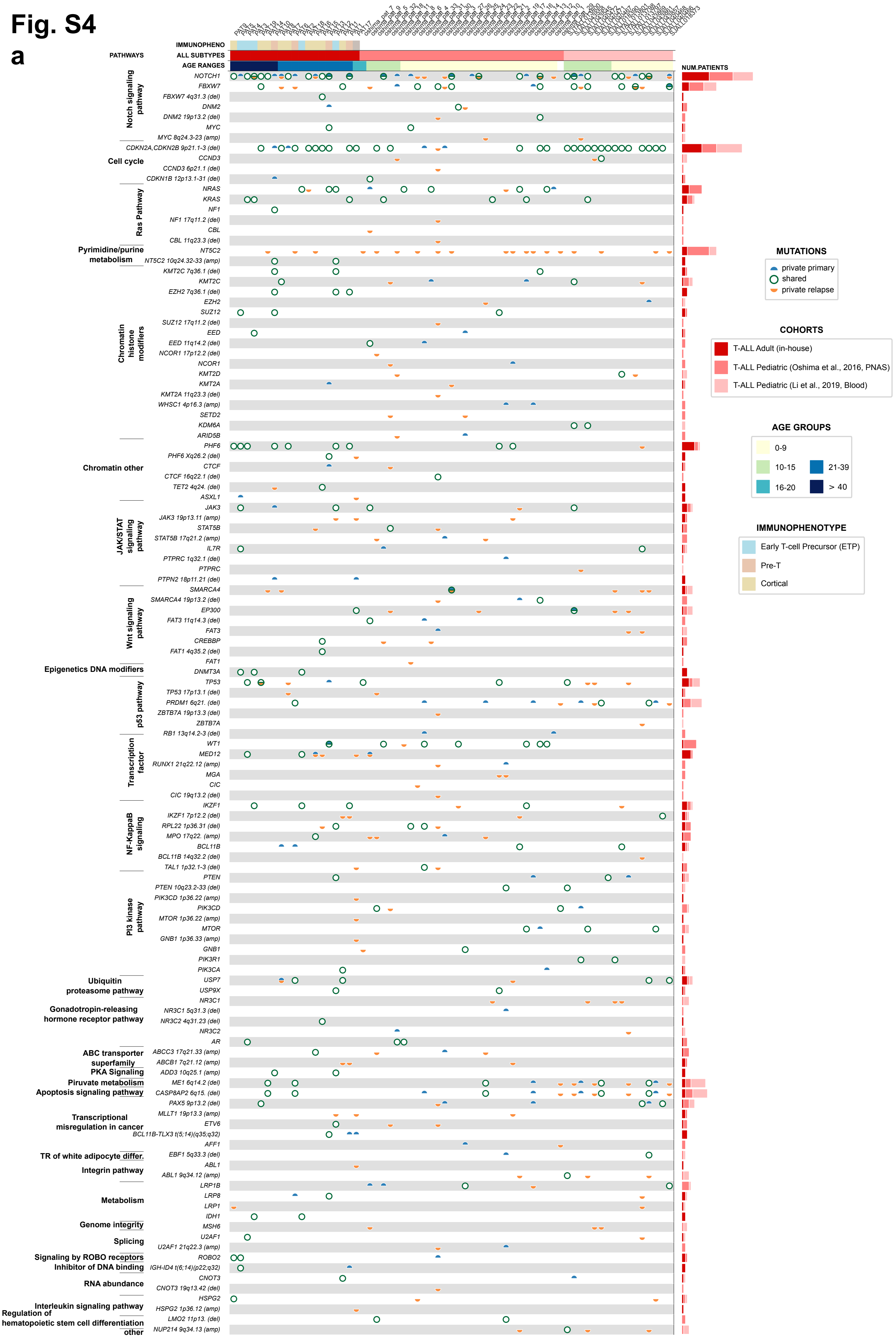

IMMUNOPHENO
PATHWAYS
ALL SUBTYPES
AGE RANGES
NUM.PATIENTS
Notch signaling pathway
NOTCH1
FBXW7
FBXW7 4q31.3 (del)
DNM2
DNM2 19p13.2 (del)
MYC
MYC 8q24.3-23 (amp)
CDKN2A,CDKN2B 9p21.1-3 (del)
Cell cycle
CCND3
CCND3 6p21.1 (del)
CDKN1B 12p13.1-31 (del)
Ras Pathway
NRAS
KRAS
NF1
NF1 17q11.2 (del)
CBL
CBL 11q23.3 (del)
Pyrimidine/purine metabolism
NT5C2
NT5C2 10q24.32-33 (amp)
KMT2C 7q36.1 (del)
KMT2C
EZH2 7q36.1 (del)
EZH2
SUZ12
SUZ12 17q11.2 (del)
EED
EED 11q14.2 (del)
NCOR1 17p12.2 (del)
NCOR1
KMT2D
KMT2A
KMT2A 11q23.3 (del)
WHSC1 4p16.3 (amp)
SETD2
KDM6A
ARID5B
Chromatin histone modifiers
PHF6
PHF6 Xq26.2 (del)
CTCF
CTCF 16q22.1 (del)
TET2 4q24. (del)
ASXL1
Chromatin other
JAK3
JAK3 19p13.11 (amp)
STAT5B
STAT5B 17q21.2 (amp)
IL7R
PTPRC 1q32.1 (del)
PTPRC
PTPN2 18p11.21 (del)
JAK/STAT signaling pathway
SMARCA4
SMARCA4 19p13.2 (del)
EP300
FAT3 11q14.3 (del)
FAT3
CREBBP
FAT1 4q35.2 (del)
FAT1
Wnt signaling pathway
Epigenetics DNA modifiers
DNMT3A
TP53
TP53 17p13.1 (del)
PRDM1 6q21. (del)
ZBTB7A 19p13.3 (del)
ZBTB7A
p53 pathway
RB1 13q14.2-3 (del)
WT1
MED12
RUNX1 21q22.12 (amp)
MGA
CIC
CIC 19q13.2 (del)
Transcription factor
IKZF1
IKZF1 7p12.2 (del)
RPL22 1p36.31 (del)
MPO 17q22. (amp)
BCL11B
BCL11B 14q32.2 (del)
TAL1 1p32.1-3 (del)
NF-KappaB signaling
PTEN
PTEN 10q23.2-33 (del)
PIK3CD 1p36.22 (amp)
PIK3CD
MTOR 1p36.22 (amp)
MTOR
GNB1 1p36.33 (amp)
GNB1
PIK3R1
PIK3CA
PI3 kinase pathway
Ubiquitin proteasome pathway
USP7
USP9X
NR3C1
Gonadotropin-releasing hormone receptor pathway
NR3C1 5q31.3 (del)
NR3C2 4q31.23 (del)
NR3C2
AR
ABC transporter superfamily
ABCC3 17q21.33 (amp)
ABCB1 7q21.12 (amp)
PKA Signaling
ADD3 10q25.1 (amp)
Piruvate metabolism
ME1 6q14.2 (del)
Apoptosis signaling pathway
CASP8AP2 6q15. (del)
PAX5 9p13.2 (del)
Transcriptional misregulation in cancer
MLLT1 19p13.3 (amp)
ETV6
BCL11B-TLX3 t(5;14)(q35;q32)
AFF1
TR of white adipocyte differ.
EBF1 5q33.3 (del)
Integrin pathway
ABL1
ABL1 9q34.12 (amp)
LRP1B
Metabolism
LRP8
LRP1
IDH1
Genome integrity
MSH6
Splicing
U2AF1
U2AF1 21q22.3 (amp)
Signaling by ROBO receptors
ROBO2
Inhibitor of DNA binding
IGH-ID4 t(6;14)(p22;q32)
RNA abundance
CNOT3
CNOT3 19q13.42 (del)
HSPG2
Interleukin signaling pathway
HSPG2 1p36.12 (amp)
Regulation of hematopoietic stem cell differentiation
LMO2 11p13. (del)
other
NUP214 9q34.13 (amp)
MUTATIONS
private primary
shared
private relapse
COHORTS
T-ALL Adult (in-house)
T-ALL Pediatric (Oshima et al., 2016, PNAS)
T-ALL Pediatric (Li et al., 2019, Blood)
AGE GROUPS
0-9
10-15
16-20
21-39
> 40
IMMUNOPHENOTYPE
Early T-cell Precursor (ETP)
Pre-T
Cortical

Fig. S4. Mutations in driver genes in primary and relapse T-ALL tumors of three cohorts

Rows are driver genes in T-ALL (collected from the literature or identified across these cohorts using IntOGen; see Supp. Methods) grouped by protein family, biological process or pathway. Columns are ALL samples grouped by cohort, and sorted by age. We added immunophenotypic information for the in-house T-ALL cohort. Primary-private and relapse-private mutations are represented as blue and yellow semicircles, respectively. Shared mutations are represented as green circles. The total number of patients affected by mutations of each gene across the three cohorts are indicated by stacked bars at the right-side of the graph. Calls from the X chromosome in the pediatric cohorts are not included (only in adults).

Fig. S5

a

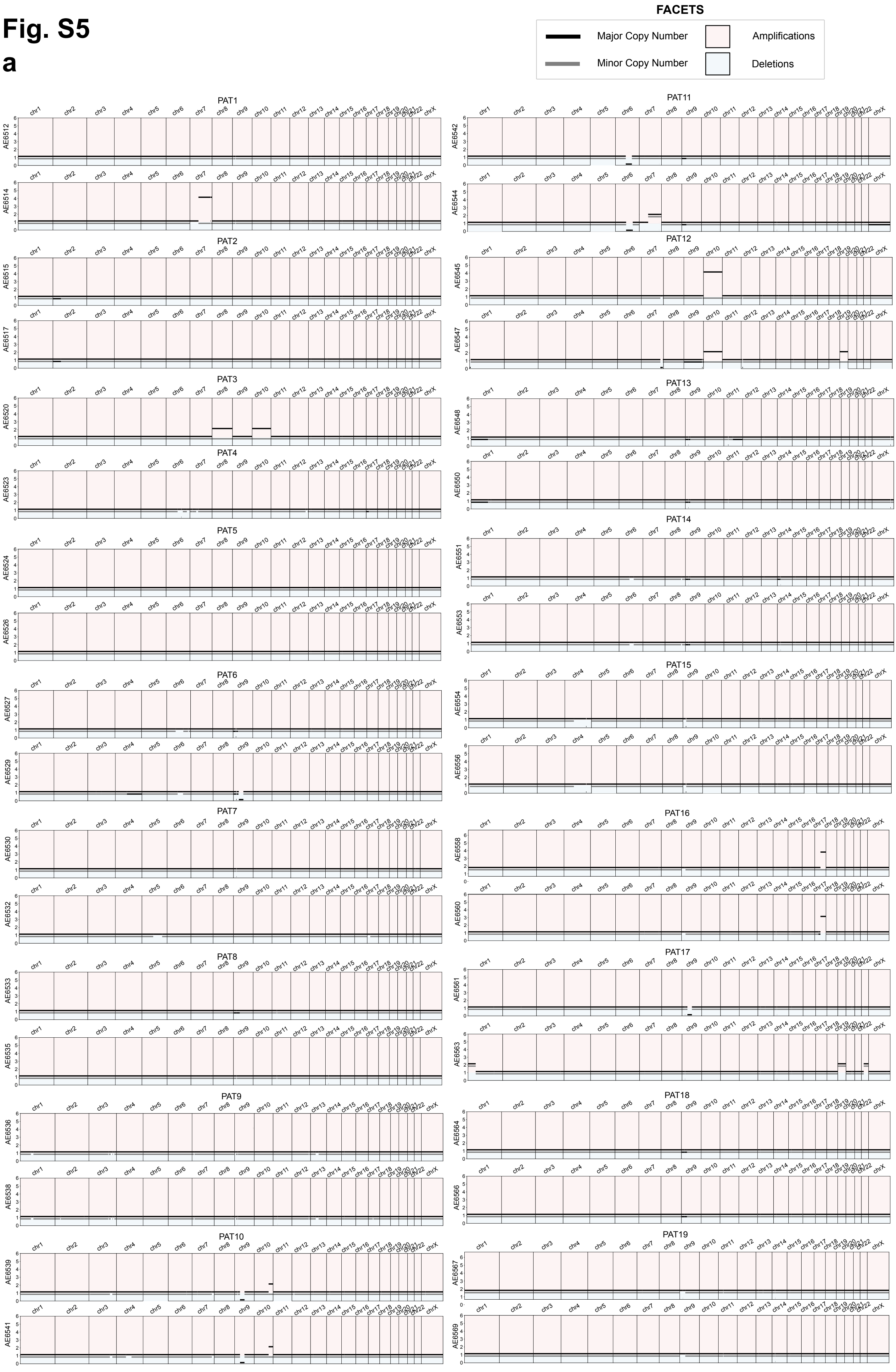
FACETS
Major Copy Number
Minor Copy Number
Amplifications
Deletions
PAT1
AE6512
AE6514
PAT2
AE6515
AE6517
PAT3
AE6520
PAT4
AE6523
PAT5
AE6524
AE6526
PAT6
AE6527
AE6529
PAT7
AE6530
AE6532
PAT8
AE6533
AE6535
PAT9
AE6536
AE6538
PAT10
AE6539
AE6541
PAT11
AE6542
AE6544
PAT12
AE6545
AE6547
PAT13
AE6548
AE6550
PAT14
AE6551
AE6553
PAT15
AE6554
AE6556
PAT16
AE6558
AE6560
PAT17
AE6561
AE6563
PAT18
AE6564
AE6566
PAT19
AE6567
AE6569

Fig. S5. Copy Number Variants detected in primary and relapse T-ALLs in the in-house cohort

In each panel, that corresponds to one T-ALL sample in the cohort, chromosomes are represented in the x-axis, with their copy number in the y-axis. Copy number of the major allele is represented as a black line and that of the minor allele as a grey line. In diploid segments, both the black and grey lines appear close to 1 (total sum of 2) but not overlapping for visual purposes. Red and blue shaded backgrounds represent amplifications and deletions, respectively. For all patients (indicated in the graph title), the top and bottom plots correspond to the primary and relapse samples, respectively.

Fig. S6

a

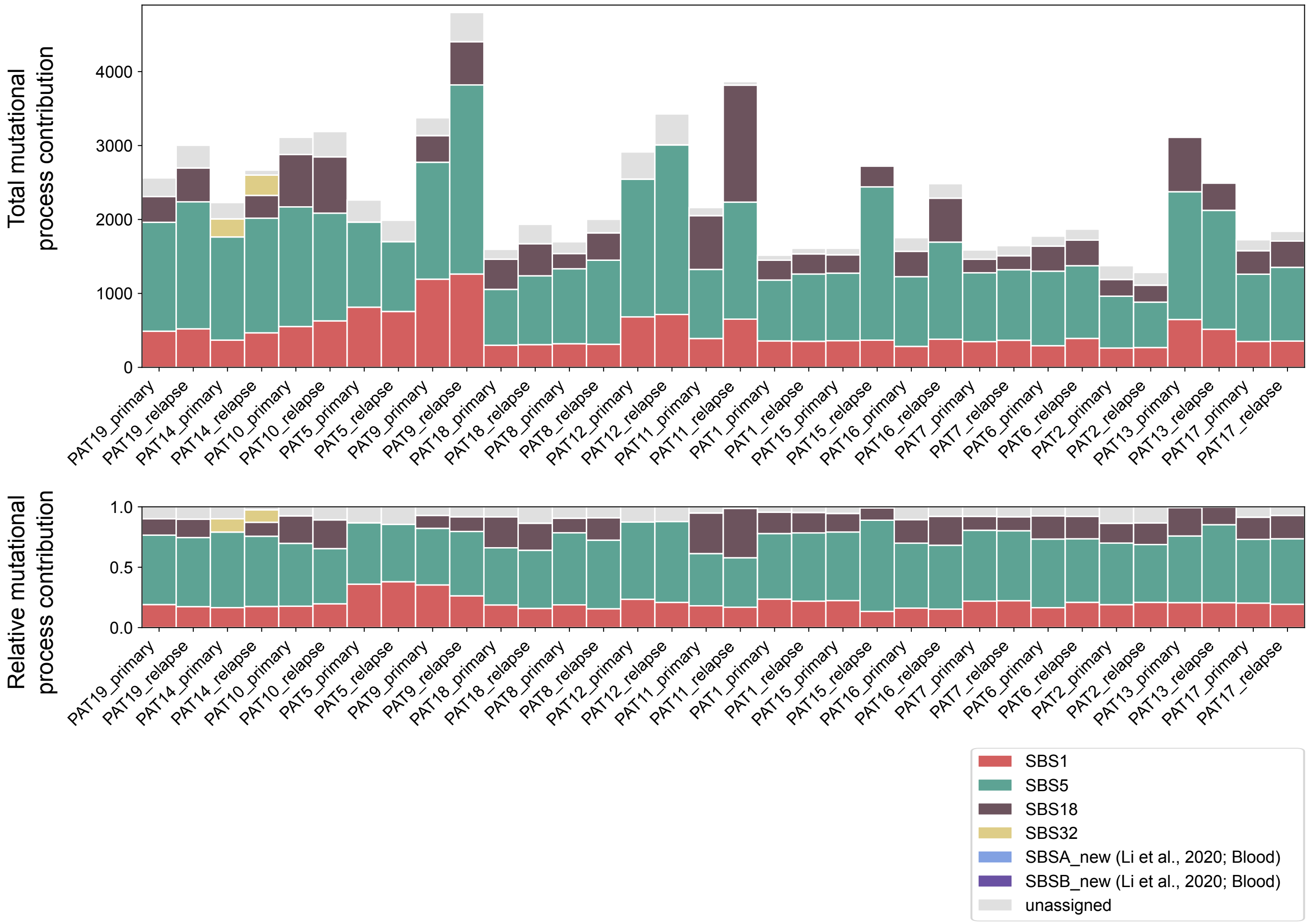

Total mutational process contribution
0
1000
2000
3000
4000
Relative mutational process contribution
0.0
0.5
1.0
PAT19_primary
PAT19_relapse
PAT14_primary
PAT14_relapse
PAT10_primary
PAT10_relapse
PAT5_primary
PAT5_relapse
PAT9_primary
PAT9_relapse
PAT18_primary
PAT18_relapse
PAT8_primary
PAT8_relapse
PAT12_primary
PAT12_relapse
PAT11_primary
PAT11_relapse
PAT1_primary
PAT1_relapse
PAT15_primary
PAT15_relapse
PAT16_primary
PAT16_relapse
PAT7_primary
PAT7_relapse
PAT6_primary
PAT6_relapse
PAT2_primary
PAT2_relapse
PAT13_primary
PAT13_relapse
PAT17_primary
PAT17_relapse
SBS1
SBS5
SBS18
SBS32
SBSA_new (Li et al., 2020; Blood)
SBSB_new (Li et al., 2020; Blood)
unassigned

Fig. S6. No mutational footprints attributable to treatments are detected

Panels represent the absolute (top) and relative (bottom) contribution of mutational processes (signatures listed in legend) to the mutation burden of the primary and relapse malignancy of each patient. As indicated in the legend, colors of the bars indicate the signatures used in the fitting process. Note that, although included in the fitting, the mutational signatures (or footprints) of drugs used in ALL treatment (recently identified in pediatric relapse tumors; [57]) are not detected in relapse T-ALLs in the in-house cohort.

Fig. S7

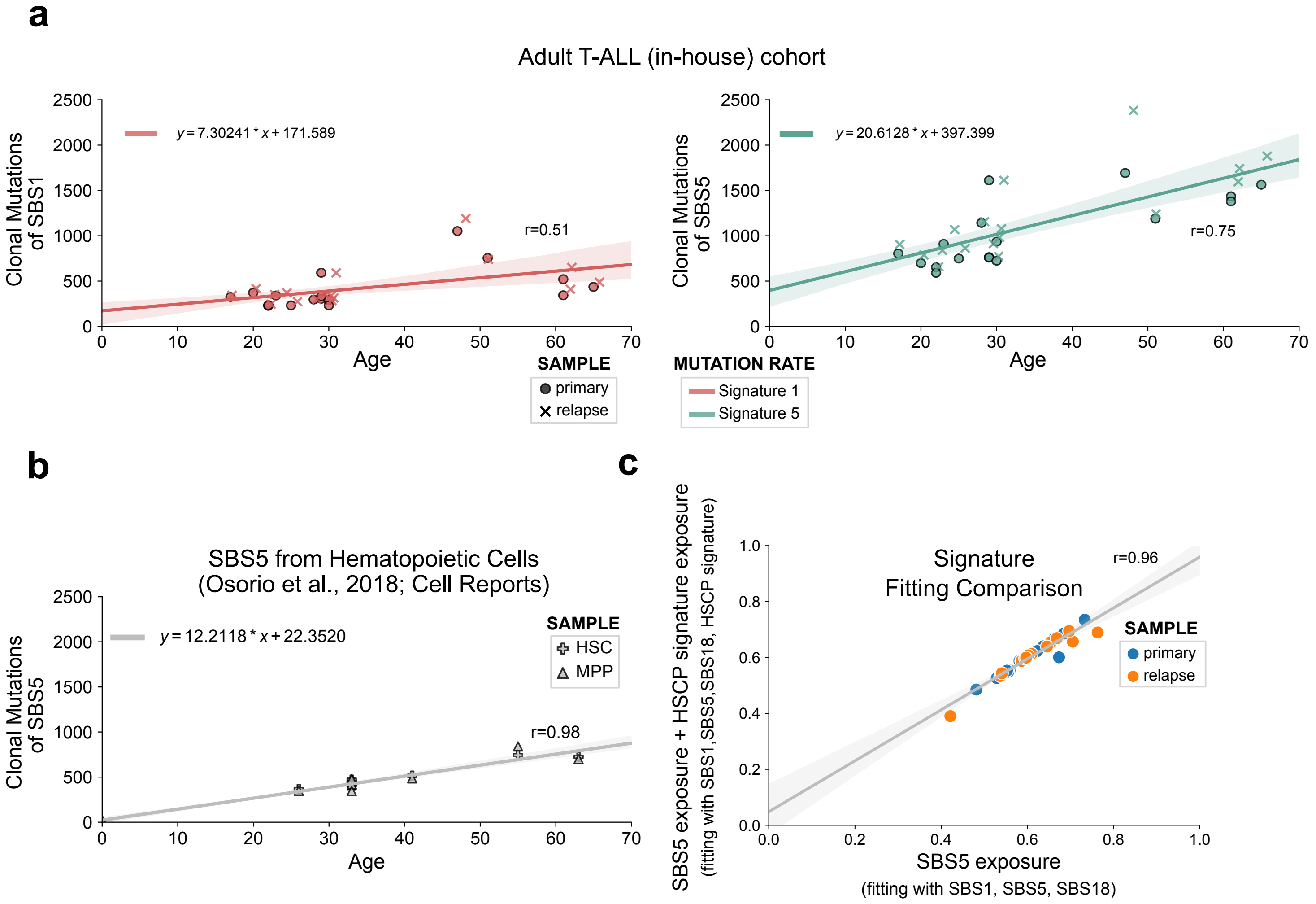

a
Adult T-ALL (in-house) cohort
Clonal Mutations of SBS1
y = 7.30241 * x + 171.589
r=0.51
Age
Clonal Mutations of SBS5
y = 20.6128 * x + 397.399
r=0.75
Age
SAMPLE
primary
relapse
MUTATION RATE
Signature 1
Signature 5
b
SBS5 from Hematopoietic Cells
(Osorio et al., 2018; Cell Reports)
Clonal Mutations of SBS5
y = 12.2118 * x + 22.3520
SAMPLE
HSC
MPP
r=0.98
Age
c
Signature
Fitting Comparison
r=0.96
SAMPLE
primary
relapse
SBS5 exposure + HSCP signature exposure
(fitting with SBS1,SBS5,SBS18, HSCP signature)
SBS5 exposure
(fitting with SBS1, SBS5, SBS18)

Fig. S7. The contribution of clock-like Signature 5 to the mutation burden of T-ALLs

a) Known clock-like signatures 1 (left panel) and 5 (right panel) contribute clonal mutations at a steady rate across the lives of T-ALL patients in the in-house cohort. Although the number of clonal mutations contributed by each process significantly correlates with the patients' age (signature 1 $p=2.30x10^{-3}$, signature 5 $p=3.64x10^{-7}$), the correlation is stronger in the case of signature 5, which had been previously observed [46]. Moreover, signature 5 contributes more age-related mutations than signature 1. Dots are primary and cross relapse samples. Trendlines following the regression are added and their equations are indicated at the top right of each panel.

b,c) Signature 5 also fits well the steady accumulation of mutations in healthy hematopoietic stem cells and multipotent progenitors (HSC and MPP; Osorio et al., 2018 [47]) with aging. Signature 5 contributed mutations fit very well (r=0.98) the mutational burden of clonally expanded HSC and MPP (b), implying that it is probably the main mutational process active in these cells and that signature 5 mutations accumulate steadily over time. When a *de novo* signature extraction (rather than the signature deconstruction presented so far in this article; see Supp. Methods) is carried out on the mutational profile of HSC and MPP a specific HSC population (HSCP) signature is extracted [47,48]. The activity (fraction of contributed mutations) of this signature across HSC and MPP cells correlates very well (r=0.96) with that obtained for the fitted signature 5. This supports the idea that the HSCP signature and signature 5 represent the same underlying mutational process active in HSC and MPP.

Fig. S8

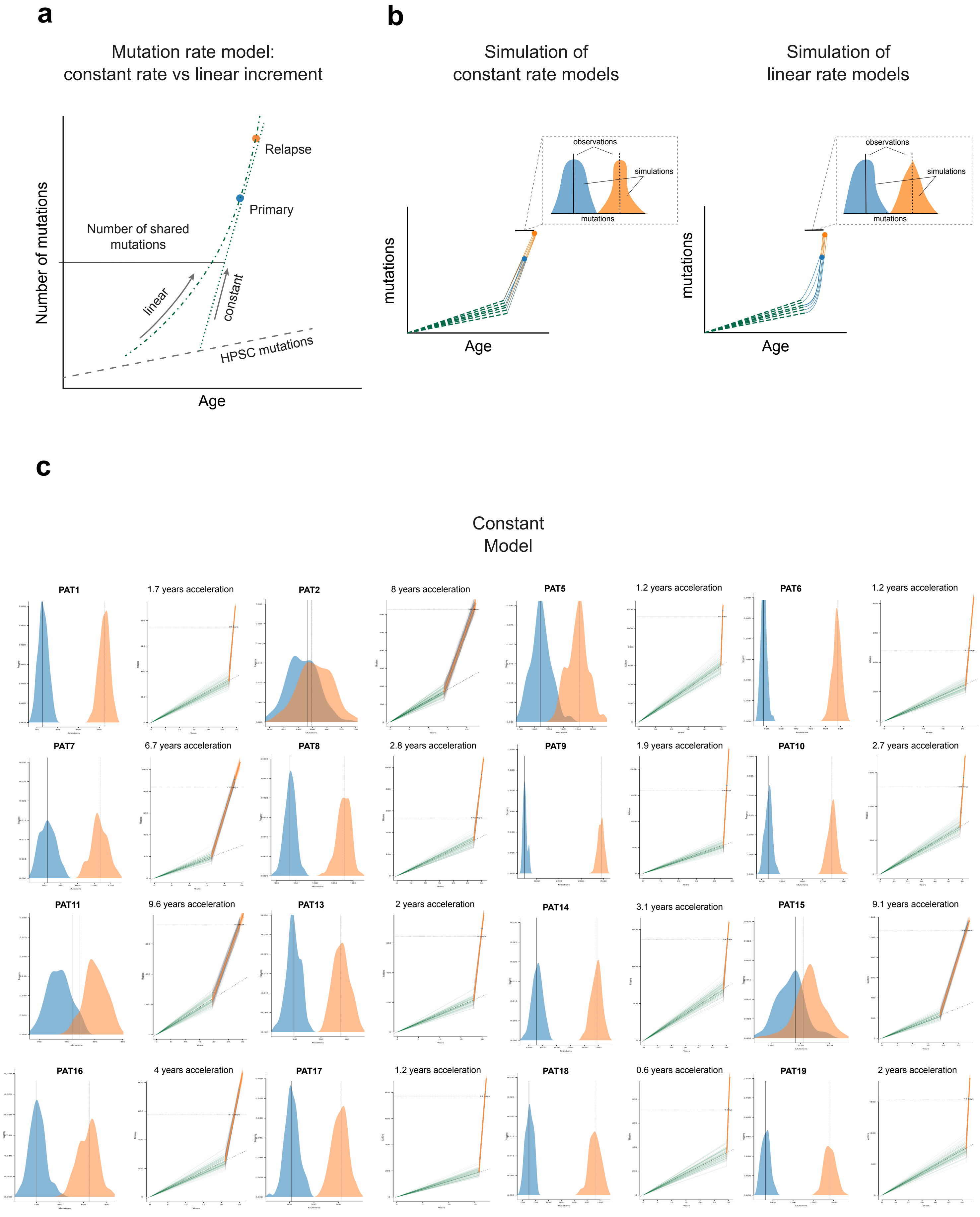

a
Mutation rate model:
constant rate vs linear increment
Number of mutations
Age
Relapse
Primary
Number of shared
mutations
linear
constant
HPSC mutations
b
Simulation of
constant rate models
Simulation of
linear rate models
observations
simulations
mutations
Age
c
Constant
Model
PAT1
1.7 years acceleration
PAT2
8 years acceleration
PAT5
1.2 years acceleration
PAT6
1.2 years acceleration
PAT7
6.7 years acceleration
PAT8
2.8 years acceleration
PAT9
1.9 years acceleration
PAT10
2.7 years acceleration
PAT11
9.6 years acceleration
PAT13
2 years acceleration
PAT14
3.1 years acceleration
PAT15
9.1 years acceleration
PAT16
4 years acceleration
PAT17
1.2 years acceleration
PAT18
0.6 years acceleration
PAT19
2 years acceleration

Linear
Model

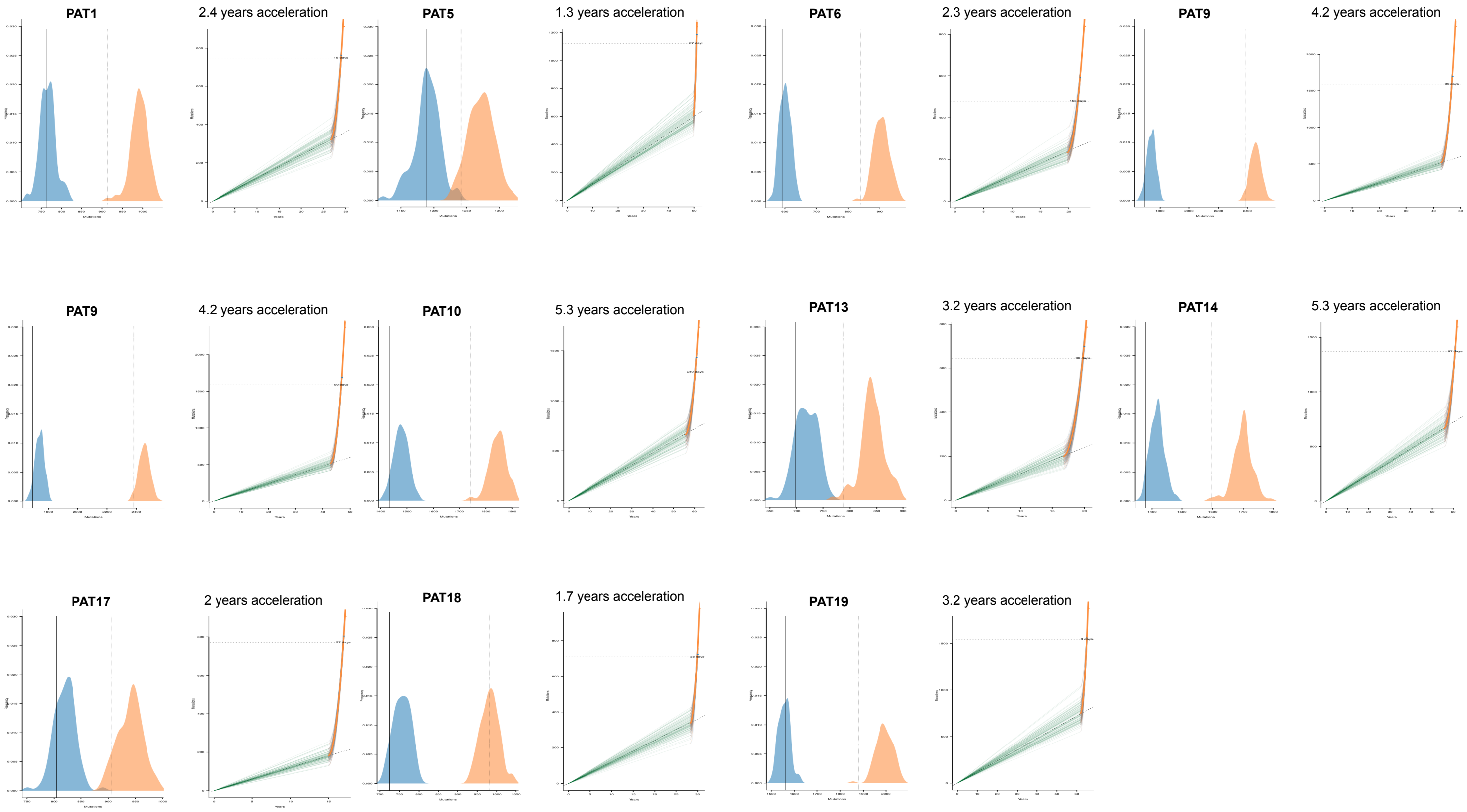

Fig. S8. Models of accelerated mutation rate in T-ALL

a) Schematic representation of two extreme models of the accelerated mutation rate in T-ALL. The baseline mutation burden increase represents the steady accumulation of mutations of HSC and MPP shown in Supplementary Figure 7. The linear model consists in a steady acceleration of the mutation rate throughout all the evolutionary history of the T-ALL. On the other hand, in the constant model the acceleration occurs only once in the evolution of the T-ALL, which after this point maintains a steady increase of the mutation burden.

b) For both, the constant (left plot) and linear (right plot) models, a number of simulations of accelerated mutation rate are carried out, represented in these schematic graphs by dotted lines. The likelihood of each explaining the observed mutation burden of primary and relapse samples is then computed, as explained in the main manuscript.

c) Real examples of the more likely models given the observed data for each patient of both types of simulations (constant and linear). The years when the mutation rate accelerated and incremented are written above each pair of graphs per patient.

Fig. S9

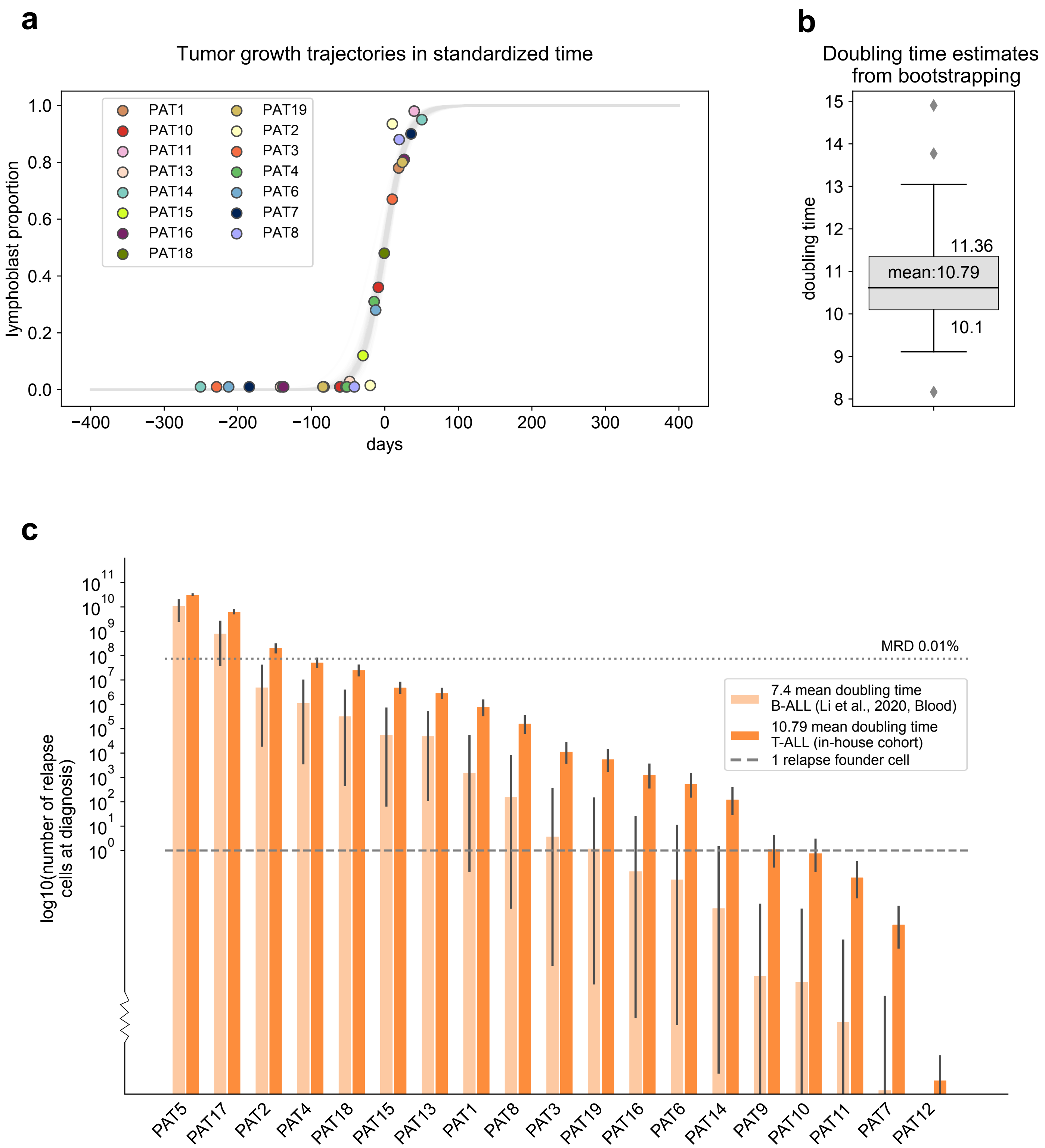
a
Tumor growth trajectories in standardized time
lymphoblast proportion
days
PAT1
PAT10
PAT11
PAT13
PAT14
PAT15
PAT16
PAT18
PAT19
PAT2
PAT3
PAT4
PAT6
PAT7
PAT8
b
Doubling time estimates from bootstrapping
doubling time
11.36
mean:10.79
10.1
c
log10(number of relapse cells at diagnosis)
MRD 0.01%
7.4 mean doubling time B-ALL (Li et al., 2020, Blood)
10.79 mean doubling time T-ALL (in-house cohort)
1 relapse founder cell
PAT5
PAT17
PAT2
PAT4
PAT18
PAT15
PAT13
PAT1
PAT8
PAT3
PAT19
PAT16
PAT6
PAT14
PAT9
PAT10
PAT11
PAT7
PAT12

Fig. S9. Estimating the doubling time of T-ALL population from the pathologists' observations

a) Bootstrapping adjustment of growth logistic curves to the counts of lymphoblasts in remission and relapse bone marrow samples carried out by the pathologist. The observations at these two points (dot pair) for each patient are represented with the same color. Paired-dates of the bone marrow sampling are re-scaled to a standardized time where 0.5 blast proportion (y-axis) falls at day 0 (x-axis) of the growth trajectory of the malignancy of each patient.

b) Boxplot with the doubling time estimates resulting from the bootstrapping. The line at the center of the boxplot is the mean. The first and third quartile of the distribution of bootstrapped doubling times are also represented.

c) Comparison of the number of relapse cells for all patients in the in-house T-ALL cohort computed at time of diagnosis using two different estimates of the doubling time of the lymphoblastic population. Light orange bars represent the size of the T-ALL relapse population computed using the doubling time estimate obtained recently for pediatric B-ALLs (Li et al., 2020) Dark orange bars represent the size of the T-ALL relapse population computed using the doubling time estimate obtained in (b). Error bars are the estimates corresponding to the doubling time in the first and third quartiles of the distribution.

Fig. S10

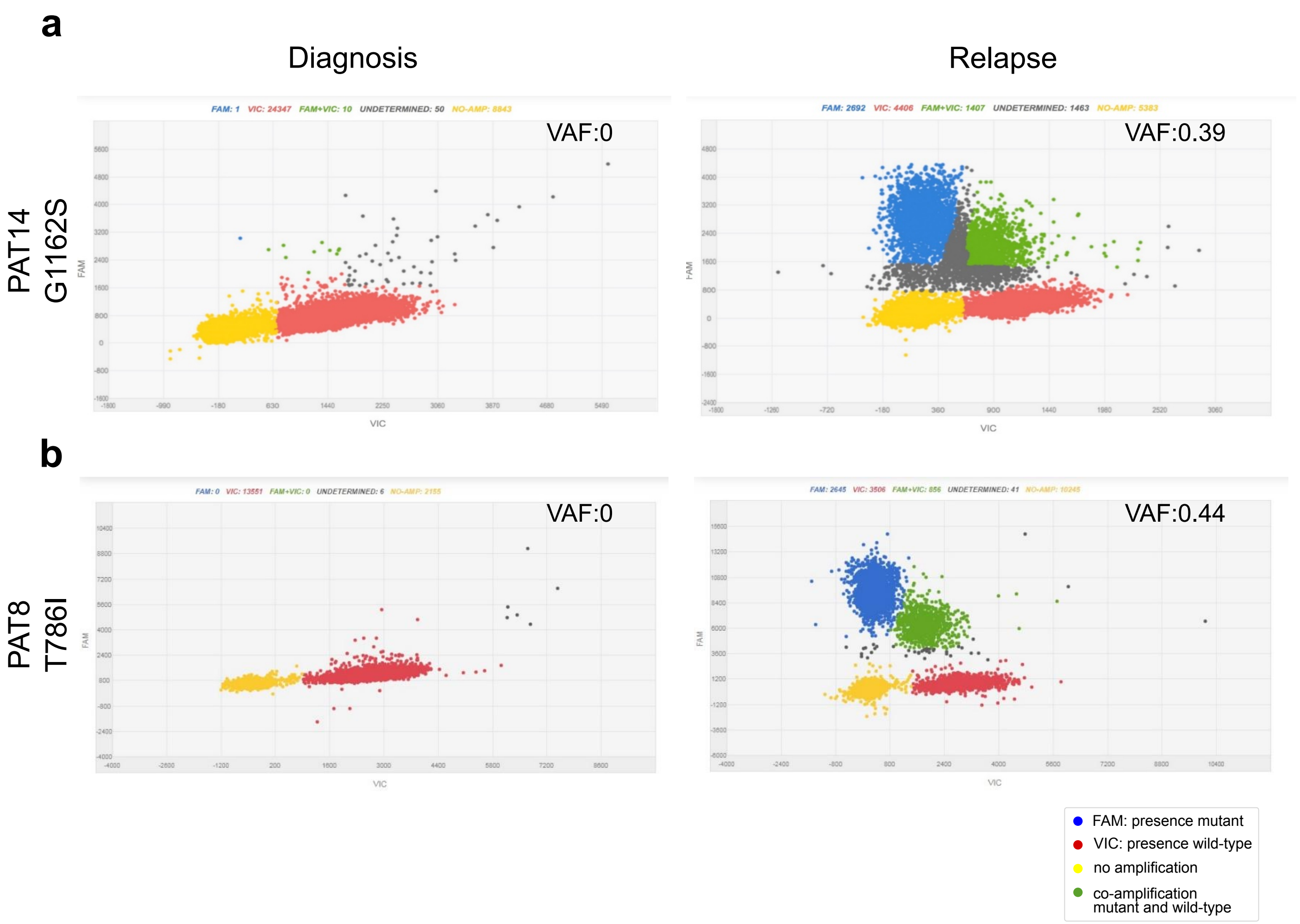
a
Diagnosis
Relapse
PAT14
G1162S
VAF:0
VAF:0.39
FAM: 1 VIC: 24347 FAM+VIC: 10 UNDETERMINED: 50 NO-AMP: 8843
FAM: 2692 VIC: 4406 FAM+VIC: 1407 UNDETERMINED: 1463 NO-AMP: 5383
b
PAT8
T786I
VAF:0
VAF:0.44
FAM: presence mutant
VIC: presence wild-type
no amplification
co-amplification mutant and wild-type

Fig. S10. Results of digital PCR on mutant *SMARCA4* in two primary samples

Detection of mutations a) G1162S and b) T786I in the relapse-enriched *SMARCA4* gene in primary samples of PAT14 and PAT8 respectively was negative in both (VAF= 0). The resolution of the dPCR in PAT14 was 0.089% whereas in PAT8 it was 0.11%. The VAF detected of the mutants in relapse derived from the dPCR is similar to the one detected by NGS which are both close to the expected 0.5 for an heterozygous variant with no normal contamination (0.39 vs 0.403 and 0.44 vs 0.346). Scatter plots showing the distribution of the data points based on the dyes used (VIC and FAM). Blue dots (FAM) represent presence of mutant *SMARCA*4 and red dots (VIC) represent wild-type *SMARCA4*; yellow refers to no amplification and green to co-amplified wild-type and mutant species.

Fig. S11

a

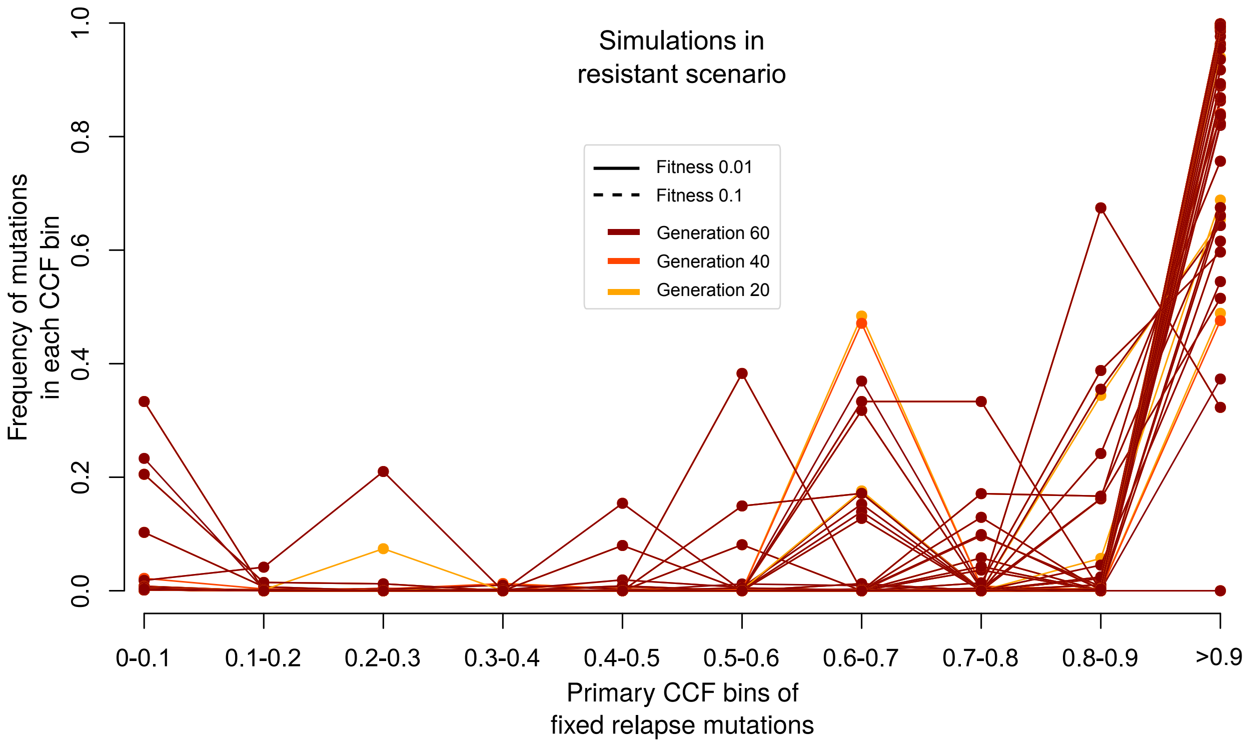

Simulations in
resistant scenario
Fitness 0.01
Fitness 0.1
Generation 60
Generation 40
Generation 20
Frequency of mutations
in each CCF bin
0.0
0.2
0.4
0.6
0.8
1.0
0-0.1
0.1-0.2
0.2-0.3
0.3-0.4
0.4-0.5
0.5-0.6
0.6-0.7
0.7-0.8
0.8-0.9
>0.9
Primary CCF bins of
fixed relapse mutations

Fig. S11. Distribution of primary CCF of relapse fixed mutations in the simulated resistant scenario

Distribution (frequency) of CCF values of mutations in synthetic primary T-ALL populations in evolutionary simulations following the resistant scenario defined in the toy example of Figure 5b. The dots represent mutations binned at different CCF values with the frequency that each bin represents with respect to all mutations in each synthetic relapse population. All the results of six simulation settings with different values of fitness of driver mutations and number of cell generations are presented.
