# Supplementary material for: The evolution of relapse of adult T cell acute lymphoblastic leukemia
Source: Genome Biol. 2020 Nov 23;21:284. doi: 10.1186/s13059-020-02192-z (PMC7682094; doi:10.1186/s13059-020-02192-z)
Supplement: Supplementary file 3 — Additional file 3. Additional methods. Some of the filtering steps have been extended for clarification in this file. [file 13059_2020_2192_MOESM3_ESM.pdf]

Supplementary Note

Preprocessing and filters of somatic mutation calls

As explained in the Methods section we have processed the mutations (SNVs and InDels) from the original VCF output of Strelka to the final MAF file of calls analyzed. The first thing we did was to filter out any mutation non-labelled as PASS or DP in the FILTER columns of their corresponding VCF. We noticed that for a few patients the number of mutations in the relapse sample was lower than the primary, contrary to what we would expect taking into account that the relapse cells had more time to accumulate mutations compared to the primary. Therefore, we decided to check whether there were mutations labellel as PASS or DP in the primary that were present in the relapse original VCF that we missed at filtering. We realized that this was the case so we decided to also do the reverse exercise and add the missed calls to the filtered set of mutations of each sample. We have called these shared mutations FISHED (see below Additional file 3: Fig. S1 a).

Another critical point was that we observed substantial differences between samples of the same patient regarding tumor burden as well as within the entire cohort. We suspected that there could be some polymorphisms within the somatic calls of the samples. We used gnomAD to annotate the variants with population frequencies and decide to filter out those with a frequency above 0.01 (see Additional file 3: Fig S1b)

The clonal classification, that is separating clonal from subclonal mutations, is explained in detail in the Methods section. In the Additional file 3: Fig S1 c we are showing the Cancer Cell Fraction (CCF, see equation below) of each mutation in the primary and relapse samples colored or shaped according to their clonal classification in the primary and relapse respectively. In almost all patients, the shared clonal mutations are a well defined blue dotted cloud of points with its centroid approximately at CCF 1 of both axis (samples).

$$CCF = \frac{VAF*(p*cn+2*(1-p))}{p}$$

being *p* the purity of the sample and *cn* the copy number of the region where the mutation falls. VAF means variant allele frequency and is calculated as follows:

$$VAF = \frac{ar}{tr}$$

where *ar* refers to reads mapping to the alternative allele and *tr* the total number of reads mapping to that particular position.

Finally, we were notified by the sequencing center (CNAG) that PAT3 and PAT4 primary samples seemed to have the DNA damaged. In this figure, PAT3 shows evidence of that by its large number of mutations and CCF values. We have not included those samples and as a consequence, these two patients were out in most of our analysis except for reporting protein affecting mutations in known ALL cancer genes of interest.

Fig. S1

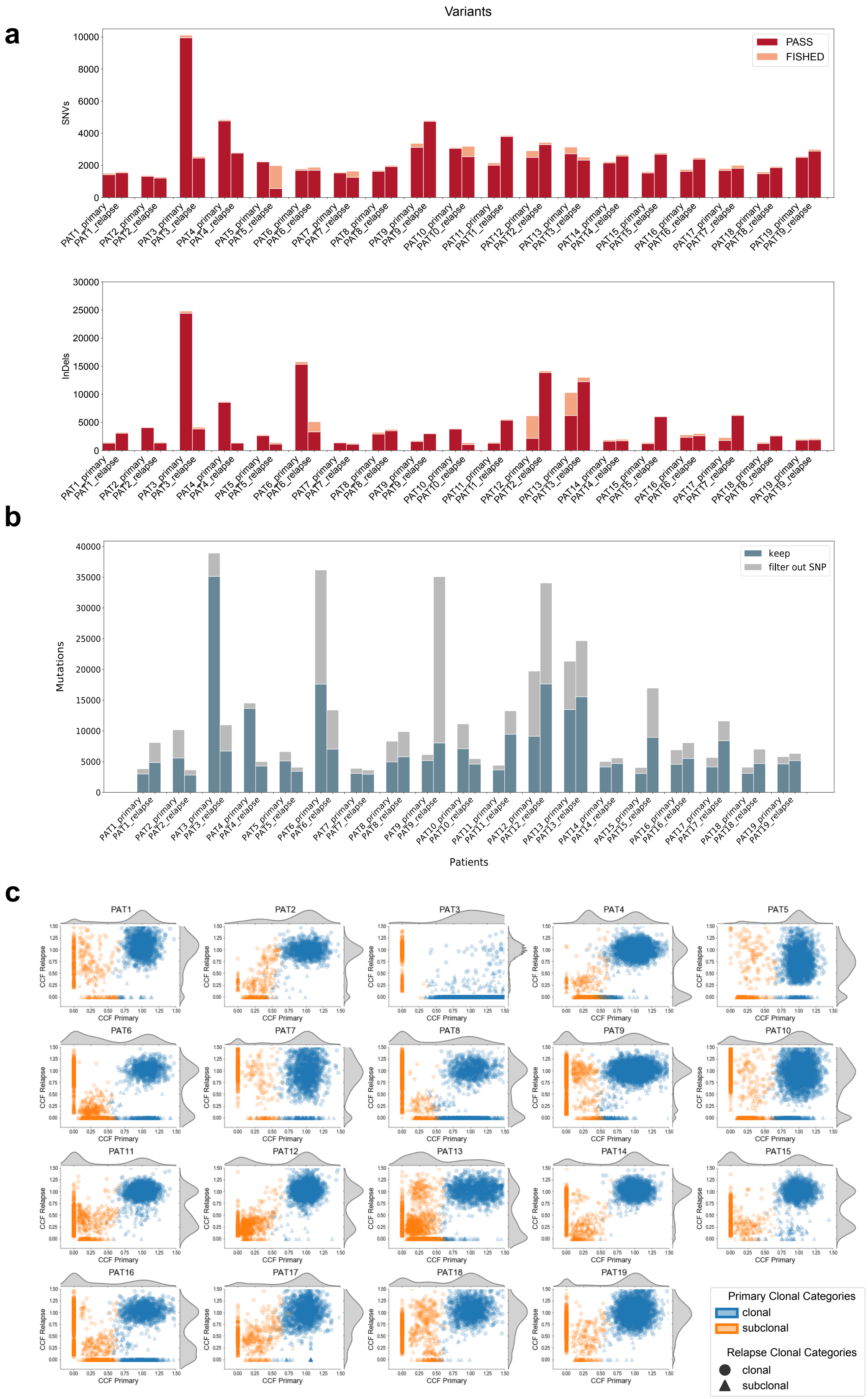
a
Variants
SNVs
PASS
FISHED
InDels
b
Mutations
keep
filter out SNP
Patients
c
PAT1
PAT2
PAT3
PAT4
PAT5
PAT6
PAT7
PAT8
PAT9
PAT10
PAT11
PAT12
PAT13
PAT14
PAT15
PAT16
PAT17
PAT18
PAT19
CCF Relapse
CCF Primary
Primary Clonal Categories
clonal
subclonal
Relapse Clonal Categories
clonal
subclonal

Fig. S1. Filter steps of mutations

a) Barplot of primary and relapse sample of each patient showing the number of SNVs (up panel) and InDels (low panel) that have the PASS label in FILTER column of the VCF (red) and the rescued shared variants called FISHED (orange). b) Barplot of primary and relapse samples of each patient showing all mutations as the proportion of mutations that were filtered out due to their high frequency (> 0.01) in the population as annotated by Gnomad and the proportion that are believed to be somatic. c) Scatterplots showing the CCF of each mutation in primary (x-axis) and relapse (y-axis). Color and shape of each data point (mutation) is indicated in the legend.
