# Supplementary material for: The evolution of relapse of adult T cell acute lymphoblastic leukemia
Source: Genome Biol. 2020 Nov 23;21:284. doi: 10.1186/s13059-020-02192-z (PMC7682094; doi:10.1186/s13059-020-02192-z)
Supplement: Supplementary file 4 — Additional file 4. Review history. [file 13059_2020_2192_MOESM4_ESM.docx]

Review History

**First round of review**

**Reviewer 1**

**Are you able to assess all statistics in the manuscript, including the appropriateness of statistical tests used?** Yes, and I have assessed the statistics in my report.

**Comments to author:**

The study of Inés Sentís used whole-genome somatic mutations of 15 primary T-ALLs from adult patients and the corresponding relapse malignancies. They analyzed mutations evolution upon treatment in comparison with 238 pediatric and young adult ALL cases. The results showed that the emergence of the relapse clone occurred several months before the diagnosis of the primary T-ALL. This suggested improving early detection of the malignancy is key to prevent its relapse. It is a great effort added to understand the mechanisms of relapse of adult T-ALL cases. This will be of broad interest to others in this field. There are several questions that need to be clarified.

General comments:

1. Only based on Fig. 1a, it is difficult to conclude that the burden of somatic point mutations of adult ALL cases tends to be higher than that of cases of the pediatric malignancy. The authors can refer to Fig. 8. of paper Liu YF, et al. EBioMedicine. 2016. PMID: 27428428.

2. In reference 57, they mentioned 12 genes which were relapse-specific somatic alterations enriched. How many of they can be identified in the sequenced 15 primary T-ALLs?

3. The authors suggested that the major clone in the relapse malignancy already present at the moment of diagnosis. Most of the conclusion is based on the estimation and simulation. If that is true, most of relapse-specific somatic alterations should been found before the start of the treatment, e.g. target deep sequence.

Minor:

1. When the author use abbreviation, it is better to have its full describe at the first time, e.g. SBS1.

2. It is not clear to me what was used as the input for the UMAP analysis in Figure1 b.

3. When calculated the Cancer Cell Fraction (CCF), how the author defined the p (the purity of the sample). This information cannot find the Table S1. Clinical Information.

**Reviewer 2**

**Are you able to assess all statistics in the manuscript, including the appropriateness of statistical tests used?** No, I do not feel adequately qualified to assess the statistics.

**Comments to author:**

Understanding somatic genetic alterations underlying T-ALL development and relapse after treatment is significantly important. The authors characterized 15 T-ALL somatic mutations from adult patients and the corresponding relapse malignancies. The authors also comprehensively analyzed the evolution upon treatment in comparison with over 200 pediatric and young adult ALL patients. The authors have found that the emergence of the relapse clone occurred a few months before the diagnosis of the primary T-ALL which suggest that improving the early detection ofT-ALL could be a key step to prevent the relapse. The data are convincing and support their conclusion.

One minor comment is that Notch and USP7 on the list are also critical genes for Treg cell development and functional stability. So it should be interesting to discuss whether the mutants of these top listed genes such as Notch and USP7 may also affect tumor-immune system interaction.

**Authors Response**

**Point-by-point responses to the reviewers’ comments:**

**Reviewer #1** : The study of Inés Sentís used whole-genome somatic mutations of 15 primary T-ALLs from adult patients and the corresponding relapse malignancies. They analyzed mutations evolution upon treatment in comparison with 238 pediatric and young adult ALL cases. The results showed that the emergence of the relapse clone occurred several months before the diagnosis of the primary T-ALL. This suggested improving early detection of the malignancy is key to prevent its relapse. It is a great effort added to understand the mechanisms of relapse of adult T-ALL cases. This will be of broad interest to others in this field. There are several questions that need to be clarified.

We thank the reviewer for appreciating the significance of our work.
General comments:

1.	Only based on Fig. 1a, it is difficult to conclude that the burden of somatic point mutations of adult ALL

cases tends to be higher than that of cases of the pediatric malignancy. The authors can refer to Fig. 8. of paper Liu YF, et al. EBioMedicine. 2016. PMID: 27428428.

We thank the reviewer for pointing us to this reference. As suggested, we have now included it in the manuscript. The modified sentence now reads.

*Nevertheless, the burden of somatic point mutations of adult ALL cases tends to be higher than that of cases of most of the subtypes of the pediatric malignancy, as has been previously observed [43] (Fig. 1a).*

2. In reference 57, they mentioned 12 genes which were relapse-specific somatic alterations enriched. How many of they can be identified in the sequenced 15 primary T-ALLs?

The 12 genes enriched for relapse-specific alterations are NR3C1, NR3C2, TP53, NT5C2, FPGS, CREBBP, MSH2, MSH6, PMS2, WHSC1, PRPS1, and PRPS2.

Of these, we found:

-	NT5C2 relapse-specific mutations in three patients: PAT6, PAT14,PAT16

- TP53 mutations in 4 patients (PAT4,PAT7,PAT13,PAT19): 2 shared mutations, 1 private

primary, 2 relapse specific

-	CREBBP with a shared mutation in PAT15

-

According to the table of Figure 1 C in Li et al., 2020 *Blood* the T-ALL subtype (yellow color columns)

presented mutations relapse-specific in NT5C2, NR3C1 and TP53.

The other relapse-enriched genes seem to be more B-ALL specific .

3. The authors suggested that the major clone in the relapse malignancy already present at the moment of diagnosis. Most of the conclusion is based on the estimation and simulation. If that is true, most of relapse-specific somatic alterations should been found before the start of the treatment, e.g. target deep sequence.

This is a very interesting suggestion by the reviewer. We have specifically looked for two SMARCA4 mutations that were identified as private-relapse in PAT8 and PAT14 in the primary samples of these two patients using digital PCR technology. Since digital PCR has a lower limit of bulk sequencing, it may be able to detect this mutation --and by extension-- the presence of the relapse clone in the primary samples.

The two mutations probed and the digital PCR limit of detection in the two samples are:

PAT8 mutation: T786I → dPCR limit of detection: aprox 0.11%
PAT14 mutation: G1162S → dPCR limit of detection: 0.089%.

The results of both dPCR were negative. The limit of detection of the digital PCR (~1/1000 cells in both samples) is not enough to detect these mutations in the primary samples.

This is clear in the updated Figure 5a (copied below), in which this limit of detection of the digital PCR has been added to the bars representing the size of the relapse population at the time of diagnosis of the primary ALLs. In both, PAT8 and PAT14, the estimated size of the population is several orders of magnitude below this limit of detection. We now describe this new result to the manuscript.

Minor:

1. When the author use abbreviation, it is better to have its full describe at the first time, e.g. SBS1.

We have carefully checked the manuscript (including figure legends) to fully describe the meaning of each acronym on its first use.

2. It is not clear to me what was used as the input for the UMAP analysis in Figure1 b.

The input of the UMAP is a matrix of 96 types of trinucleotide changes (the affected nucleotide and both the surrounding unchanged ones) times 182 WGS ALL cases across cohorts. Therefore, every cell in the matrix corresponds to the count of one type of tri-nucleotide change in one tumor. We have introduced one sentence explaining this in the new version of the manuscript.

The modified sentence reads:

*The UMAP was run on a matrix of the counts of all possible tri-nucleotide changes (96) across ALL patients of all cohorts.*

3. When calculated the Cancer Cell Fraction (CCF), how the author defined the p (the purity of the sample). This information cannot find the Table S1. Clinical Information.

As suggested, we have now included the purity of primary and relapse samples of each patient as Primary_seq_purity, Relapse_seq_purity columns in the Clinical Information Table S1.

**Reviewer #2** : Understanding somatic genetic alterations underlying T-ALL development and relapse after treatment is significantly important. The authors characterized 15 T-ALL somatic mutations from adult patients and the corresponding relapse malignancies. The authors also comprehensively analyzed the evolution upon treatment in comparison with over 200 pediatric and young adult ALL patients. The authors have found that the emergence of the relapse clone occurred a few months before the diagnosis of the primary T-ALL which suggest that improving the early detection ofT-ALL could be a key step to prevent the relapse. The data are convincing and support their conclusion.

We thank the appreciative comment by the reviewer.

One minor comment is that Notch and USP7 on the list are also critical genes for Treg cell development and functional stability. So it should be interesting to discuss whether the mutants of these top listed genes such as Notch and USP7 may also affect tumor-immune system interaction.

This is an intriguing suggestion by the reviewer. Indeed, USP7 translates in a ubiquitin-specific protease 7 which is known for deubiquitination of p53 and Notch1 preventing their degradation and stabilizing their levels [[1,2]](https://www.zotero.org/google-docs/?NsW3bi) . Inhibition and downregulation of USP7 has been proven to downregulate Notch1 protein level and to decrease leukemic cell growth in vitro and in vivo [[2,3]](https://www.zotero.org/google-docs/?UXYivo) . The oncogenic power of USP7 is to maintain Notch1 pathway active by stabilizing Notch1 active protein levels.

Also interesting is the potential interaction of the Tregs, suggested by the reviewer, with the leukemic cells and its influence in the development of the disease [4,5]. While USP7-mutant Tregs are known to be involved in several diseases (see references below), further studies are required to clarify their specific effect in T acute lymphoblastic leukemia.

We have added a mention to the possibility of USP7 mutations being another form of alteration of the NOTCH pathway in the manuscript. The modified sentence reads:

*Intriguingly, mutations affecting USP7, a known deubiquitinase of NOTCH1 were detected in 3 adult and 3 pediatric patients, raising the possibility of yet another form of alteration of the NOTCH pathway in leukemogenesis [69–71].*

1. [Richter-Pechańska P, Kunz JB, Hof J, Zimmermann M, Rausch T, Bandapalli OR, et al. Identification of a genetically defined ultra-high-risk group in relapsed pediatric T-lymphoblastic leukemia. Blood Cancer J. 2017;7.](https://www.zotero.org/google-docs/?5sIRMF)
2. [Shan H, Li X, Xiao X, Dai Y, Huang J, Song J, et al. USP7 deubiquitinates and stabilizes NOTCH1 in T-cell acute lymphoblastic leukemia. Signal Transduct Target Ther. 2018;3:29.](https://www.zotero.org/google-docs/?5sIRMF)
3. [Q J, Ca M, Km A, Y Z, Bt G-D, Kk W, et al. USP7 Cooperates with NOTCH1 to Drive the Oncogenic Transcriptional Program in T-Cell Leukemia. Clin Cancer Res. 2018;25:222–39.](https://www.zotero.org/google-docs/?5sIRMF)
4. van Loosdregt J, Fleskens V, Fu J, Brenkman AB, Bekker CP, Pals CE, Meerding J, Berkers CR, Barbi J, Gröne A, Sijts AJ, Maurice MM, Kalkhoven E, Prakken BJ, Ovaa H, Pan F, Zaiss DM, Coffer PJ. Stabilization of the transcription factor Foxp3 by the deubiquitinase USP7 increases Treg-cell-suppressive capacity. Immunity. 2013 Aug 22;39(2):259-71. doi: 10.1016/j.immuni.2013.05.018.
5. Ostroukhova M, Qi Z, Oriss TB, Dixon-McCarthy B, Ray P, Ray A. Treg-mediated immunosuppression involves activation of the Notch-HES1 axis by membrane-bound TGF-beta. J Clin Invest. 2006 Apr;116(4):996-1004. doi: 10.1172/JCI26490. Epub 2006 Mar 16.

**Second round of review**

**Reviewer 1**

The authors' responses quite satisfactory and the revised version has been much improved.
